# Metabolic syndrome among children and adolescents in low and middle income countries: a systematic review and meta-analysis

**DOI:** 10.1186/s13098-020-00601-8

**Published:** 2020-10-27

**Authors:** Zebenay Workneh Bitew, Ayinalem Alemu, Ermias Getaneh Ayele, Zelalem Tenaw, Anmut Alebel, Teshager Worku

**Affiliations:** 1 St. Paul’s Hospital Millennium Medical College, Addis Ababa, Ethiopia; 2 Ethipian Public Health Institute, Addis Ababa, Ethiopia; 3 College of Medicine and Health Sciences, Hawassa University, Hawassa, Ethiopia; 4 College of Health Science, Debremarkose University, Debre Markos, Ethiopia; 5 Australian Centre for Public and Population Health Research, School of Public Health, Faculty of Health, University of Technology Sydney, Ultimo, NSW Australia; 6 College of Health and Medical Sciences, School of Nursing and Midwifery, Haramaya University, Harar, Ethiopia

**Keywords:** Metabolic syndrome, MetS, Components of metabolic syndrome, Low and middle income countries, Lmics

## Abstract

**Background:**

Metabolic syndrome (MetS) is a clustering of cardiovascular risk factors, which is rising in the low and middle income countries (LMICs). There are various studies with inconsistent findings that are inconclusive for policy makers and program planners. Thus, this systematic review and meta-analysis aimed at estimating the pooled prevalence of MetS and its components in LMICs.

**Methods:**

Electronic searches were conducted in international databases including PubMed, Web of Science, EMBASE (Elsevier), Scopus, CINAHL (EBSCOhost), Science direct (Elsevier), Food Science and Technology Abstracts (FSTA), Global Health and Medline, and other sources (World Cat, Google Scholar, and Google). The pooled estimates were computed in the random effect model. The pooled prevalence was computed using the three diagnostic methods (IDF, ATP III and de Ferranti). Publication bias was verified using funnel plot and Egger’s regression test. Subgroup and sensitivity analysis were performed to identify the possible sources of heterogeneity among the included studies.

**Result:**

In this study, 142,142 children and adolescents from 76 eligible articles were included to compute the pooled prevalence of MetS and its components in LMCIs. MeTs among overweight and obese population was computed from 20 articles with the pooled prevalence of 24.09%, 36.5%, and 56.32% in IDF, ATP III and de Ferranti criteria, respectively. Similarly, a total of 56 articles were eligible to compute the pooled prevalence of MetS in the general population of children and adolescents. Hence, Mets was found in 3.98% (IDF), 6.71% (ATP III) and 8.91% (de Ferranti) of study subjects. Regarding the components of MetS, abdominal obesity was the major component in overweight and obese population and low HDL-C was the most common component in the general population. This study also revealed that males were highly affected by MetS than females.

**Conclusion:**

This study illustrates that MetS among children and adolescents is an emerging public health challenge in LMICs, where the prevalence of obesity is on the move. Preventive strategies such as community and school based intervention need to be designed. Promoting physical activities and healthy eating behaviors could avert this problem.

## Background

Metabolic syndrome (MetS) is a constellation of interconnected risk factors of metabolic origin leading to atherosclerotic cardiovascular diseases [1]. The common risk factors include elevated triglycerides, altered glucose metabolism, reduced high density lipoprotein cholesterol (HDL-C), and elevated blood pressure and adiposity [2]. It usually resulted from dysregulated cellular metabolism, leading to insulin resistance [3]. MetS is also associated with a multitude of disorders such as diabetic mellitus, increased uric acid level, hepatic steatosis, polycystic ovarian syndrome, and obstructive sleep apnea [4–8].

There are various diagnostic methods for MetS in children and adolescents. According to the International Diabetes Federation (IDF), MetS is diagnosed if children aged between 10–16 years have central adiposity (≥ 90th) and two of the followings: triglycerides (TG) ≥ 150 mg/dl, HDL-C < 40 mg/dl, systolic blood pressure (BP) ≥ 130 mmHg or diastolic BP ≥ 85 mmHg, fasting plasma glucose (FG) ≥ 100 mg/dl or previously diagnosed type 2 diabetes [9]. Based on the WHO criteria, MetS is diagnosed when three or more of the following features are found: body mass index (BMI): > 95th percentile, hyperinsulinemia or impaired fasting glucose or impaired glucose tolerance, BP > 95th percentile, TG > 105/136 mg/dL (1.2/1.5 mmol/L) for children aged < 10 and > 10 years respectively, HDL-C < 35 mg/dL (0.9 mmol/L) [10]. Adult Treatment Panel III (ATPIII) criteria modified for age defines the presence of MetS when three of the following criteria are met: TG ≥ 110 mg/dl, HDL-C ≤ 40 mg/dl, systolic BP or diastolic BP ≥ 90th, WC ≥ 90th percentile for age and gender. percentile for age and gender and FG ≥ 110 mg/dl [11]. In accordance with de Ferranti et al. MetS is clustering of at least three of the following criteria: FG ≥ 110 mg/dl, HDL-C ≤ 50 mg/dl (except in boys aged 15 to 19 years in whom the cut point is 45 mg/dl), TG ≥ 100 mg/dl, systolic BP > 90th percentile for gender, age and height, WC > 75th percentile for age and gender [12], whereas Cook et al. depicted that MetS is diagnosed when there or more of the following criteria are met: WC ≥ 90th percentile, FG ≥ 110 mg/dL (≥ 6.1 mmol/L), TG ≥ 110 mg/dL, HDL-C ≤ 40 mg/dL (1.03 mmol/L) and BP ≥ 90th percentile [13].

The prevalence of MetS in children and adolescents remains unclear [14]. However, a previous review revealed that it ranged from 0.2 to 38.9%, with a median of 3.3% (range, 0–19.2) in the general population and relatively higher in overweight (11.9%) and obese (29.2%) children [14–16]. These reports depicted that MetS in children and adolescents is increasingly becoming a major public health concern [17]. Accordingly, study findings concerning MetS among children and adolescents reported in low and middle-income countries (LMICs), are highly inconsistent and varied across countries. For instance, it is estimated to be as high as 22% in Iranian children and adolescents with sizable variations among the diagnostic methods [18].

A previous study has outlined that plenty of factors, primarily related to lifestyle [19], are significantly associated with an increased incidence of MetS. Consumption of fructose in the form of soft drinks, juice, and baked goods remarkably upsurge in the past four decades, which contributed to the emergence of obesity, the main predictor of MetS in children and adolescents [20–22]. It has significantly increased since 1980 contributing to 6–39% of MetS in children and adolescents [23]. Currently, obesity is one from the three global syndemics along with undernutrition and climate change, affecting both children and adults globally [24]. This problem is increasing alarmingly in developing countries due to the recent nutritional and demographic transitions [25].

Evidence-based systematic reviews are essential to inform program planners and policy-makers. However, to the best of our knowledge, systematic reviews in this area are minimal, especially in LMICs. Therefore, the main purpose of this systematic review and meta-analysis was to determine the pooled prevalence of MetS in children and adolescents in LMICs using different diagnostic methods. The findings will be very informative for policy-makers and program planners in designing preventive strategies accordingly. The results will also have a particular implication for developing countries, where the triple burden of malnutrition prevails [26]. Besides, this study will be decisive to design preventive measures for non-communicable diseases (NCDs) in the LMICs, where the trend of NCDs is increasing.

## Methods

### Eligibility criteria and information sources

In this systematic review and meta-analysis, studies conducted in LMICs with an objective of assessing the prevalence of MetS among children and adolescents were included. The studies were assessed using study area, study setups, title, abstract, and full texts prior to inclusion in this study. This study was prepared based on the Preferred Reporting Items for Systematic Reviews and Meta-analysis (PRISMA) guideline [27]. In the present study, published articles, surveys, and unpublished articles that were conducted in English were explored and included accordingly. Besides, the reference lists of included articles were checked for additional studies. Observational studies reporting the prevalence of MetS among children and adolescents conducted both in clinical and community based setups were included. Studies published until July, 2020 were searched.

However, articles with incomplete diagnostic methods and which were not fully accessible were excluded. The corresponding authors of the primary studies were communicated by email before the decision of exclusion was made. Conference proceedings and qualitative studies were also excluded. The EndNote X8 reference manager was used to manage retrieved articles.

### Search strategy and study selection

A comprehensive systematic literature search was conducted by three investigators (ZWB, AA, and TW), independently. During the searching process, we consulted a senior librarian working at St. Paul’s Hospital Millennium Medical College, Ethiopia A literature search for available articles published in English was performed using the following databases: PubMed, Web of Science, EMBASE (Elsevier), Scopus, CINAHL (EBSCOhost), Science direct (Elsevier), Food Science and Technology Abstracts (FSTA), Global Health and Medline, up to July 2020. The grey literature sources (World Cat, Google Scholar, and Google) were also explored to find out additional articles. Searching was conducted using the following key terms: (a) *population* (children, adolescent, child, school age); (b) *exposure* (associated factors, risk factors, determinants, predictors) (c) *outcome* (metabolic syndrome, MetS, components of metabolic syndrome); (d) *study design* (cohort studies, cross sectional studies, epidemiology, observational, national health surveys); (e) *study setting* (school, community based surveys, health institutions) and (f) *location* (low and middle-income countries, LMICs, developing countries, names of low and middle income countries). The Boolean search operators such as “OR”, “AND” were used during the searching process. Key terms were verified for appropriateness prior to actual searching. Example of search string in PubMed **(**Table 1**).**

**Table 1:** Search string used for searching articles from Pubmed

Population	(Children) OR (school children)) OR ("Child"[Mesh])) OR ("Adolescent"[Mesh])
Outcome	("Prevalence"[Mesh] AND "epidemiology" [Subheading]) AND ("Metabolic Syndrome"[Mesh])
Study region/country	(low and middle income countries)) OR "Afghanistan"[Mesh]) OR ("Burkina Faso"[Mesh])) OR ("Burundi"[Mesh])) OR ("Central African Republic"[Mesh])) OR ("Chad"[Mesh])) OR ("Democratic Republic of the Congo"[Mesh])) OR ("Eritrea"[Mesh])) OR ("Ethiopia"[Mesh])) OR ("Gambia"[Mesh])) OR ("Guinea"[Mesh])) OR ("Guinea-Bissau"[Mesh])) OR ("Haiti"[Mesh])) OR ("Democratic People's Republic of Korea"[Mesh])) OR ("Liberia"[Mesh])) OR ("Madagascar"[Mesh])) OR ("Malawi"[Mesh])) OR ("Mali"[Mesh])) OR ("Mozambique"[Mesh])) OR ("Niger"[Mesh])) OR ("Rwanda"[Mesh])) OR ("Sierra Leone"[Mesh])) OR ("Somalia"[Mesh])) OR ("South Sudan"[Mesh])) OR ("Sudan"[Mesh])) OR ("Syria"[Mesh])) OR ("Tajikistan"[Mesh])) OR ("Togo"[Mesh])) OR ("Uganda"[Mesh])) OR ("Yemen"[Mesh])) OR ("Angola"[Mesh]))) OR "Bangladesh"[Mesh]) OR ("Benin"[Mesh])) OR ("Bhutan"[Mesh])) OR ("Bolivia"[Mesh])) OR ("Cabo Verde"[Mesh])) OR ("Cambodia"[Mesh])) OR ("Cameroon"[Mesh])) OR ("Comoros"[Mesh])) OR ("Congo"[Mesh])) OR ("Cote d'Ivoire"[Mesh])) OR ("Djibouti"[Mesh])) OR ("Egypt"[Mesh])) OR ("El Salvador"[Mesh])) OR ("Eswatini"[Mesh])) OR ("Ghana"[Mesh])) OR ("Honduras"[Mesh])) OR ("India"[Mesh])) OR ("Kenya"[Mesh])) OR ("Micronesia"[Mesh])) OR ("Kyrgyzstan"[Mesh])) OR ("Lesotho"[Mesh])) OR ("Mauritania"[Mesh])) OR ("Moldova"[Mesh])) OR ("Mongolia"[Mesh])) OR ("Morocco"[Mesh])) OR ("Myanmar"[Mesh])) OR ("Nepal"[Mesh])) OR ("Nicaragua"[Mesh])) OR ("Nigeria"[Mesh])) OR ("Pakistan"[Mesh])) OR ("Papua New Guinea"[Mesh])) OR ("Philippines"[Mesh])) OR ("Sao Tome and Principe"[Mesh])) OR ("Senegal"[Mesh])) OR ("Melanesia"[Mesh])) OR ("Sri Lanka"[Mesh])) OR ("Tanzania"[Mesh])) OR ("Timor-Leste"[Mesh])) OR ("Tunisia"[Mesh])) OR ("Ukraine"[Mesh])) OR ("Uzbekistan"[Mesh])) OR ("Vanuatu"[Mesh])) OR ("Vietnam"[Mesh])) OR ("Middle East"[Mesh])) OR ("Zambia"[Mesh])) OR ("Zimbabwe"[Mesh])) OR ("Albania"[Mesh])) OR ("American Samoa"[Mesh])) OR ("Argentina"[Mesh])) OR ("Armenia"[Mesh])) OR ("Azerbaijan"[Mesh])) OR ("Republic of Belarus"[Mesh])) OR ("Belize"[Mesh])) OR ("Bosnia and Herzegovina"[Mesh])) OR ("Botswana"[Mesh])) OR ("Brazil"[Mesh])) OR ("Bulgaria"[Mesh])) OR ("China"[Mesh])) OR ("Colombia"[Mesh])) OR ("Costa Rica"[Mesh])) OR ("Cuba"[Mesh])) OR ("Dominica"[Mesh])) OR ("Dominican Republic"[Mesh])) OR ("Dominican Republic"[Mesh])) OR ("Equatorial Guinea"[Mesh])) OR ("Ecuador"[Mesh])) OR ("Fiji"[Mesh])) OR ("Gabon"[Mesh])) OR ("Georgia (Republic)"[Mesh])) OR ("Grenada"[Mesh])) OR ("Guatemala"[Mesh])) OR ("Guyana"[Mesh])) OR ("Indonesia"[Mesh])) OR ("Iran"[Mesh])) OR ("Iraq"[Mesh])) OR ("Jamaica"[Mesh])) OR ("Jordan"[Mesh])) OR ("Kazakhstan"[Mesh])) OR ("Kosovo"[Mesh])) OR ("Lebanon"[Mesh])) OR ("Libya"[Mesh])) OR ("Malaysia"[Mesh])) OR ("Indian Ocean Islands"[Mesh])) OR ("Mexico"[Mesh])) OR ("Montenegro"[Mesh])) OR ("Namibia"[Mesh])) OR ("Republic of North Macedonia"[Mesh])) OR ("Paraguay"[Mesh])) OR ("Peru"[Mesh])) OR ("Russia"[Mesh])) OR ("Samoa"[Mesh])) OR ("Serbia"[Mesh])) OR ("South Africa"[Mesh])) OR ("Saint Lucia"[Mesh])) OR ("Suriname"[Mesh])) OR ("Thailand"[Mesh])) OR ("Tonga"[Mesh])) OR ("Turkey"[Mesh])) OR ("Turkmenistan"[Mesh])) OR ("Venezuela"[Mesh]))
Filters	Filters: Free full text, Observational Study, in the last 10 years, Humans, English, Child: 6–12 years, Adolescent: 13–18 years

### Data extraction process

Three authors (ZWB, AA, and EGA) extracted data from included articles using a standardized data extraction form. First, the data were stored in Microsoft excel, 2016 by two authors (ZWB, AA, and EGA), independently. Next, the data were cleaned and made ready for the final analysis using the excel spreadsheet. Finally, the data were exported to the STATA software for analysis. The data extraction format included: name of the author (s), publication year, study country, sample sizes, age of the study population, population group, MetS with diagnostic methods, and components of MetS. Discrepancies between the authors were solved through discussion and consensus, and with active involvement of the other author (ZT) (Additional file 1).

### Quality assessment of studies

Two authors (ZWB & AA) independently assessed the quality of included studies using a Joanna Briggs Institute (JBI) Critical Appraisal Checklist for Observational Studies [28]. The tool has four options (Yes, No, Unknown, and Not Applicable). One is given for yes and zero for other options. The minimum score was zero and the maximum was eight. The scores were summed up and changed to percentages. Studies with quality scores of > 50% were included in this meta-analysis (Additional file 2). The mean scores of the two reviewers were used for final decision of inclusion of the studies in this systematic review and meta-analysis. During critical appraisal, the author (ZT) participated actively in solving differences between the two authors.

### Summary measures

The primary outcome of this study was the prevalence of MetS among children and adolescents in LMICs using various diagnostic methods. The pooled prevalence of MetS was calculated in the general population and overweight and/or obese children and adolescents separately. The general population includes underweight, normal weight, overweight and obese children and adolescents. The other outcomes were components of metabolic syndromes, the prevalence of MetS based on country, continent, and economic level of countries, where the original studies were done. Based on economic level, LMICs were further divided in to low income economies (LIE), lower middle income economies (LMIE), and upper middle income economies (UMIE) [29]. The pooled prevalence of MetS was also computed among males and females. The prevalence was calculated by dividing the total number of events (MetS) to the total sample size and multiplying it by 100. The binomial distribution formula was used to compute the standard error for each original study. The “metan” commands were used to compute the pooled estimates using STATA (version 15) software. The pooled estimates were presented with their 95% CIs. The effect sizes were prevalence of MetS in LMICs and the respective components of MetS.

### Statistical methods and analysis

In the current meta-analysis, STATA Version 15 (STATA Corporation, College Station Texas) software was used for computing the pooled estimates. The pooled estimates were computed using both random and fixed effect models. Due to the presence of high heterogeneity among studies, the pooled estimates were computed using random-effects models and were weighted using the inverse variance method. Subgroup analyses were performed using different parameters. The pooled estimates in the general and overweight and/or obese population were presented separately. For the subgroup analysis, data were extracted based on study continent, study county, the economic level of the study countries, type of diagnosis and gender of study subjects. The appropriateness of each datum was verified before the analyses. The pooled estimates were presented with their 95% CIs. Likewise, the heterogeneities among the included studies in the pooled estimates were presented with I^2^ test statistic and P-value. The results of meta-analyses were presented using forest plot, summery tables, and texts.

### Publication bias and heterogeneity

Publication bias was assessed using the funnel plot asymmetry and Egger’s regression test at a 5% significant level [30]. Heterogeneity among included studies was explored using forest plot, I^2^ test, and the Cochrane Q statistics [31]. The I^2^ values of 25%, 50%, and 75% were interpreted as low, medium, and high heterogeneity, respectively [32]. In this meta-analysis, significant heterogeneity was considered when the I^2^ value was ≥ 50%, with P-value < 0.05. The possible sources of significant heterogeneity were addressed through sub-group and sensitivity analyses.

## Results

### Selection of eligible studies

**Fig. 1:**
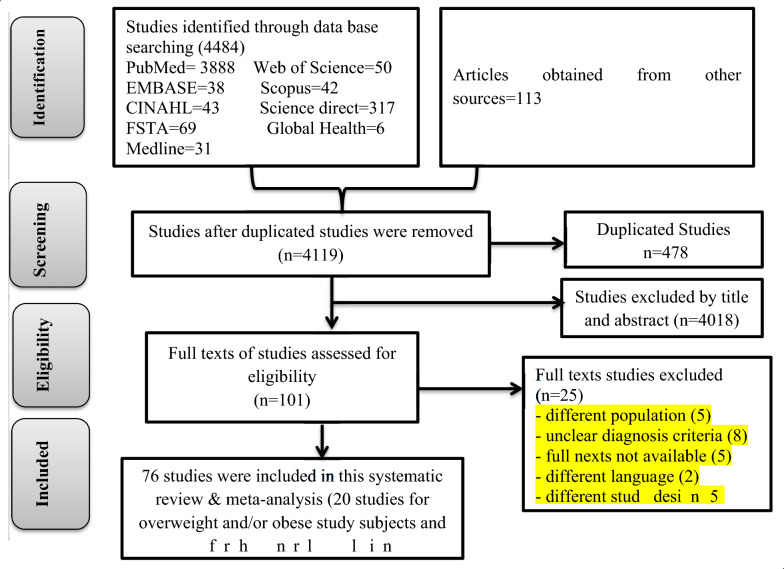
PRISMA flow chart showing study selection process

A total of 4597 articles were obtained in the initial search. After removal of 478 due to duplicates, 4119 were remained and screened for titles and abstracts. Following this, 4018 studies were removed after reading titles and abstracts. The full texts of 101 articles were downloaded and assessed for eligibility criteria. Twenty five studies were excluded due to the following exclusion criteria: different study population, no full test, unclear diagnostic criteria, letter to editor, written in non-English language, and different study design (Additional file 3). Finally, 76 articles [33–108] were included in the final analysis in this meta-analysis (Fig. 1).

### Characteristics of the included studies

All studies included in this study were cross-sectional studies. Regarding study population, 20 studies [35, 47, 50, 55, 57, 58, 63, 69, 71, 72, 77, 79, 84, 87–89, 91, 92, 95, 104] were conducted among overweight and/or obese children and adolescents, and 56 studies [33, 34, 36–46, 48, 49, 51–54, 56, 59–62, 64–68, 70, 73–76, 78, 80–83, 85, 86, 90, 93, 94, 96–103, 105–108] were conducted among the general population of children and adolescents. This review included 142,142 study participants from 76 articles. Of which, 138,236 were the general population, whereas 3906 were overweight and obese population. The sample size of included studies ranged from 51 in Tunisia [58] to 37,504 in Brazil [52]. The age of study population across the included studies ranged between 5 to 20 years. Most of the studies were conducted in UMIE Asian countries and very few articles were found from Africa. The quality of articles was also assessed using the JBI checklist, and 56 articles had medium quality. The remaining 20 studies had high quality (Tables 2, 3).

**Table 2:** Characteristics of studies used to compute the prevalence of metabolic syndrome in LMICs in overweight/obese adolescents

Author, year	Country	Sample size	Prevalence of MetS		Age	MetS with diagnostic methods N (%)			Components of Mets (%)					Qaulity scores
			M (%)	F (%)		IDF	ATP-III	de.F	Ab. obesity	Low HDL	High TGL	High FG	High BP	
Dejavitte et al. 2020 [1]	Brazil	354	142 (15.5)	212 (5.7)	10–19	34 (9.6)	–	–	77.4	49.4	5.6	15	1.1	8
Cornejo-Monthedoro et al. 2017 [2]	Peru	273	143 (19.6)	130 (25.4)	10–15	61 (22.3)	–	–	81.7	63.7	29.7	5.9	5.1	8
Rinaldi et al. 2016 [3]	Brazil	147	71 (12.7)	76 (7.9)	6–10	–	15 (10.2)	–	47.6	24.5	23.8	0.8	11.6	8
Vukovic et al. 2015 [4]	Serbia	199	84 (33)	115 (29.6)	4–19	62 (31.2)	–	–	9.1	45.3	15.7	4.3	34.6	6
Medina et al. 2015 [5]	Mexico	137	67 (28.4)	70 (17)	6–12	–	31 (22.6)	–	56.9	34.3	46	0.73	21.1	6
Damak et al. 2015 [6]	Tunisia	51	28 (21)	23 (22)	15–18	11 (21.6)	–	-	58.8	9.8	–	27.4	58.8	6
Tavares Giannini et a 2014 [7]	Brazil	163	52	111	10–18	16 (9.8)	33 (20.2)	–	85.9	42.3	29.4	–	13.5	8
			52	111					70.5	23.9	8.6	1.8	18.4	
Gobato et al. 2014 [8]	Brazil	79	40 (52.8)	39 (47)	10–18	36 (45.5)	–		–	–	–	–	–	6
Casavalle et al. 2014 [9]	Argentina	139	78	61	8–14	–	30 (21.6)	–	55.4	29.5	31.7	1.5	25.2	6
Yee et al.2013 [10]	Myanmar	46	25	21	5–12	9(19.6)	–	–	54.4	60.9	13.0	4.3	8.7	6
Sewaybrickera et al. 2013 [11]	Brazil	65	32 (29.1)	33 (33.3)	10–18	18 (27.7)	19 (29.2)	–	27.7	27.7	27.7	27.7	27.7	5
			32 (25)	33 (33.3)	10–18				27.7	29.2	29.2	27.7	29.2	
Rizzo et al. 2013 [12]	Brazil	321	147 (18.4)	174 (18.4)	10–16	59 (18.3)	–	–	55	35.5	18.5	2	21	6
Saffari et al. 2012 [13]	Iran	100	42 (57)	58 (67)	6–16	–	63 (63)	–	81	70	74	12	36	5
Jamoussi t al, 2012 [14]	Tunisia	186	49 (40.8)	137 (32)	6–18	64 (34.4)	–	–	100	27	15	51	28	5
Cua et al. 2012 [15]	Philippines	350	206 (20)	144 (18)	10–18	67 (19)	–	–	98	17	24	12	25	6
Costa et al. 2012 [16]	Brazil	121	62	59	10–14	48 (39.7)	62 (51.2)	90 (74.4)	81	54.5	16.5	7.4	54.5	6
			62	59	10–14				81	54.5	34.7	1.7	76	
			62	59	10–14				96.7	92.6	40.5	1.7	76	
Hassan et al. 2011 [17]	Egypt	462	144	288	7–18	–	–	184 (39.7)	85.7	32	42.9	13.9	30.3	6
Panamonta et al. 2010 [18]	Thailand	186	–	–	10–15	6 (3.2)	–	–	–	10.2	28.0	1.1	8.6	6
Juárez-López etal, 2010 [19]	Mexico	466	272 (21)	194 (20)	11–13	93 (20)	–	–	49	69	29	4	13	6
Caceres et al. 2008 [20]	Bolivia	61	30 (40)	31 (32)	5–18	–	22 (36)	–	100	55.7	42.6	8.2	24.5	6

**Table 3:** Characteristics of studies included to compute the prevalence of metabolic syndrome in low and middle income countries

Author, year	Country	Sample size	Prevalence in Males (%)	Prevalence in Females (%)	Age	MetS with diagnostic method N (%)			Population		Gender (%)		Components of Mets (%)					Quality score
						IDF	ATP-III	de.Ferranti	Non-OB	OW/OB	M	F	Ab. Obesity	Low HDL	High TGL	High FG	High BP	
Zhu et al. 2020 [1]	China	15045	7711 (2.8)	7334 (1.7)	7–18	346 (2.3)	–	–	–	–	1.4	0.9	21.8	14.4	5.5	3	3.7	6
Mahajan et al. 2020 [2]	India	296	128 (3.9)	168 (3.6)	14–19	–	11 (3.7)	–	–	–	1.7	2.1	9.8	64.9	6.4	0.3	16.9	8
Bekele et al. 2020 [3]	Ethiopia	824	403 (10.2)	421 (14.5)	13–19	102 (12.4)	–	–	6.3	6.1	5	7.4	32.2	20.6	26.2	57.8	8.5	6
Ahmadi et al. 2020 [4]	Iran	1035	456 (9.6)	579 (6)	6–18	79 (7.6)	–	–	–	–	4.3	3.3	27.8	56.2	7.4	9.1	8	8
Zhao et al. 2019 [5]	China	1766	871 (4)	895 (2)	10–15	59 (3.3)	–	–	0.1	3.2	2	1.3	30	78 4	10	11	7	8
Zhang et al.2019 [6]	China	683	366 (6.6)	317 (3.5)	8–15	–	35 (5.1)	–	0.1	5	3.5	1.6	–	–	–	–	–	6
Wang et al. 2019 [7]	China& Spain	2126	1011	1115	10–15	30 (1.4)	–	–	–	–	–	–	16.7	15.8	5.5	4.1	12.6	8
Oliveira et al. 2019 [8]	Brazil	1035	470 (5.2)	565 (3.9)	12–20	47 (4.5)	–	–	3.4	1.1	2.4	2.1	14.9	26.4	4.2	4.4	9.0	6
Suebsamran et al. 2018 [9]	Thailand	393	152 (5.9)	241 (1,2)	13–16	12 (3.1)	23 (5.8)	44 (11.2)	0.3	2.8	2.3	0.8	15.6	25.6	3.3	0.8	4.6	6
			152 (10.5)	241 (2.9)	13–16				1	4.8	4.1	1.7	15.6	28.4	13.7	0.2	11.7	
			152(15.8)	241(8.3)	13–16				3.1	8.1	6.1	5.1	34.6	51.3	17.8	0.2	11.7	
Gupta et al. 2018 [10]	India	2100	1149 (4.4)	951 (9)	10–16	69 (3.3)	74 (3.5)	–	–	–	2.4	0.9	8.0	16.9	9.2	13.5	7.6	6
Dos Santos et al. 2018 [11]	Brazil	274	88 (5)	186 (4.4)	12–18	13 (4.7)	–	–	–	–	1.8	2.9	15.3	25.2	6.6	5.1	8.8	6
Andaki et al.2018 [12]	Brazil	1480	707 (12.6)	773 (8.5)	6–10	–	–	99 (6.7)	–	–	6	0.7	27.5	43	10.7	0.7	10.7	6
Sekokotla et al. 2017 [13]	S.Africa	371	116 (6)	255 (3.1)	13–18	15 (4)	–	–	–	–	1.9	2.1	30	28.8	8.6	4.6	32.6	6
Wang et al. 2016 [14]	China	1770	857 (1.4)	913 (0.8)	7–17	19 (1·1)	–	–	–	–	0.68	0.42	11.9	11.6	5.5	1.6	0.8	6
Suarez-Ortegón et al. 2016 [15]	Colombia	494	256 (8.6)	238 (8.8)	5–9	–	–	43(8.7)	–	–	4.5	4.2	33	47.6	20.4	4	2.6	6
Kuschnir et al. 2016 [16]	Brazil	37504	15006 (2.9)	22498 (2.4)	12–17	975 (2.6)	–	–	–	–	1.2	1.4	12.6	32.7	4.6	4.1	8.2	6
Karandish et al. 2016 [17]	Iran	1749	886 (8)	863 (2.9)	10–16	–	96 (5.5)	–	–	–	4.1	1.4	9.2	25	31.2	17	22.8	8
de Carvalho et al. 2016 [18]	Brazil	421	170	251	9–19	17 (4.1)	–	–	–	–	–	–	8.6	26.1	20.9	0.5	11.9	6
Ramı´rez-Ve´ lez et al. 2016 [19]	Colombia	1922	877 (0.11)	1045 (.48)	9–17	6 (0.3)	119 (6.2)	211 (11)	0.15	0.15	0.04	0.26	–	–	–	–	–	6
			877	1045	9–17				4	2.2	2.5	3.7	–	–	–	–	–	
			877	1045	9–17				7	4	4.5	6.5	–	–	–	–	–	
Rosini et al. 2015 [20]	Brazil	1011	481 (13)	530 (15)	6–14	–	143 (14.1)	–	3	11.1	6.2	7.9	30.4	37.6	26.1	11.6	13.6	8
Bhat et al. 2015 [21]	India	899	311 (3.8)	588 (3.5)	10–18	14(1.5)	32 (3.6)	–	1.7	1.9	1.4	2.2	3.7	17	31	9.8	4	6
Bhalavi et al. 2015 [22]	India	405	182 (7.7)	223 (11.7)	10–19	–	40 (9.9)	–	9.9	–	3.5	6.4	2.2	58.3	27.9	13.8	22.4	6
Bortoloti et al. 2015 [23]	Brazil	683	301	382	11–17	–	37 (5.4)	–	–	–	–	–	3.5	44.7	18.6	0.6	7	6
Reyes et al. 2014 [24]	Venezuela	916	450 (3.11)	466 (1.3)	9–18	14 (1.5)	20 (2.2)	–	–	–	1.5	0.7	10.2	8.6	10.5	3.6	8.7	6
			450	466	9–18				–	–	–	–	9.5	31.4	7.5	3.6	0.7	
Rerksuppaphol et al. 2014 [25]	Thailand	348	189 (3.7)	159 (4.4)	–	–	–	14 (4)	0.6	3.4	2	2	29.6	–	12.6	8.9	18.4	6
Rashidi et al. 2014 [26]	Iran	2246	1113 (11)	1133 (7)	10–19	–	203(9)	–	6.1	2.9	5.5	3.5	10.3	24.1	33.5	16.4	22.1	6
Pitangueira et al. 2014 [27]	Brazil	502	213(16.4)	289 (10)	7–14	–	64 (12.8)	–	2.8	10	7	5.8	26.7	52.8	41.8	7.2	29.1	6
Mbowe et al. 2014 [28]	Guatemala	302	144	158	8–13	–	6(2)	–	–	–	–	–	12.3	17.2	43.4	1.7	2.0	8
Li et al. 2014 [29]	China	910	485 (10.9)	425 (3.8)	11–16	69 (7.6)	–	–	–	–	5.8	1.8	22.5	46.8	9.7	6.3	16.9	8
Fadzlina et al. 2014 [30]	Malaysia	1014	387 (3.4)	627 (2.1)	13	26 (2.6)	–	–	–	2.6	1.3	1.3	17.3	6.3	6.6	3.5	4.9	6
Wang et al. 2013 [31]	China	2564	1279 (0.4)	1285 (6.7)	10–18	140 (5.5)	331 (12.9)	–	–	2.1	3.4	2.1	31.4	14.1	10.3	12.6	9.9	6
			1279 (1.0)	1285 (24.7)	10–18				0.5	12.4	8.1	4.8	32.6	11.9	25.3	12.6	19.4	
Tandona et al. 2013 [32]	India	695	346	349	10–18	118 (17)	137 (19.7)	–	0.2	16.8	–	–	39.3	27.3	37	13.2	14	6
Sua´ rez-Ortego’n et al. 2013 [33]	Colombia	1461	718 (1)	743 (1.3)	10–16	18 (1.2)	37 (2.5)	124 (8.5)	0.4	0.8	0.5	0.7	8.8	26.8	6.9	4.5	3.6	6
			718	743					–	–	–	–	22.2	54.6	27.5	0.7	6	
			718	743					–	–	–	–	8.8	29.6	20.3	0.7	8.6	
Singh et al. 2013 [34]	India	1160	658 (3.84)	502 (1.6)	10–18	–	31 (2.67)	–	0.9	1.7	2.2	0.47	5.66	10.66	3.44	6.3	2.75	8
Sarrafzadegan et al. 2013 [35]	Iran	1992	1014	978	–	90 (4.5)	–	240 (12.1)	–	–	–	–	9	24.9	10.9	4.6	22.8	6
			1014 (13.7)	978 (10.3)	–				–	–	7	5.1	21	24.9	42.9	4.6	22.8	
Qorbani et al.2013 [36]	Iran	3565	1793 (2.3)	1772 (2.9)	10–18	91 (2.6)	–	–	–	1.2	1.4	–	–	–	–	–	–	6
Khashayar et al. 2013 [37]	Iran	5738	2863	2875	10–18	144 (2.5)	–	–	1.1	1.4	–	–	16.3	24.9	6.5	12.1	5.4	8
Andrabi et al. 2013 [38]	India	758	385 (3.9)	373 (3.8)	8–18	–	29 (3.8)	–	0.4	3.4	2	1.8	4.5	4.4	3.8	1.3	–	8
Xu et al. 2012 [39]	China	8764	4495 (0.7)	4269 (0.5)	7–11	52 (0.6)	–	–	0.05	0.55	0.35	0.25	13.6	5.2	3.9	2.1	1.8	8
Nasreddine et al. 2012 [40]	Lebanon	263	112	115	–	24 (9.1)	26 (9.9)	–	0.4	8.7	–	–	50.6	38.4	10.6	4.9	12.2	6
Mehrkash et al. 2012 [41]	Iran	450	225 (4.4)	225 (1.6)	15–18	–	15 (3.3)	–	0.9	2.4	2.4	0.9	4.2	11.6	33.3	12.4	4.9	6
Chen et al. 2012 [42]	China	3814	–	–	10–18	372 (9.8)	–	–	0.2	9.6	–	–	–	–	45	13	–	6
Liu e al 2010 [43]	China	1844	938 (5.7)	906 (7.5)	7–14	–	121 (6.6)	–	1.9	4.7	2.9	3.7	23.4	15.8	16.1	0.2	23.5	6
Khader et al. 2010 [44]	Jordan	512	235	277	10–18	11 (2.1)	–	–	–	–	–	–	5.8	26.1	17.2	7.2	6.2	6
Hirschler et al. 2010 [45]	Argentina	1009	508 (5.3)	501 (6)	6–14	–	57 (5.8)	–	0.4	5.4	2.8	3	27.6	19.7	12.9	0.8	8.5	6
Ella et al. 2010 [46]	Egypt	4250	1806 (7.4)	2444 (7.4)	10–18	–	308 (7.2)	–	–	–	3.1	4.1	20	24	22	4	25.5	8
Afkhami-Ardekani et al. 2010 [47]	Iran	932	402	530	10–19	75 (8)	63 (6.7)	–	–	–	–	–	–	–	–	–	–	5
Seki et al. 2009 [48]	Brazil	2170	1103 (4.2)	1067 (3)	6–16	–	78 (3.6)	–	0.3	3.3	2.1	1.5	11.2	43.2	6.4	0.6	9.8	8
Salem et al., 2009 [49]	Iran	1221	–	1221 (3.9)	11–18	–	48 (3.9)	–	–	–	–	3.9	1.2	44.7	15.8	7.9	1.5	8
Mirhosseini et al. 2009 [50]	Iran	622	–	622 (6.5)	15–17	–	40 (6.5)	–	4.8	1.7	–	6.5	3.7	57	24.5	16.7	6.1	5
Matsha et al. 2009 [51]	S.Africa	1272	496 (8.1)	776 (5.5)	10–16	24 (1.9)	83 (6.5)	–	2.2	4.3	3.1	3.4	9.9	48.3	9.3	4.2	9.3	5
			496(3.4)	776(0.9)					0.95	0.95	1.3	0.6	10.8	48.3	4.1	4.2	6.8	
Li et al. 2008 [52]	China	2761	1478 (3.4)	1283 (4)	15–19	–	–	102 (37)	2.2	1.5	1.8	1.9	3·8	53·8	19·6	0·8	18·2	6
Singh et al. 2007 [53]	India	1083	571 (3.2)	512 (5.5)	12–17	–	46 (4.2)	–	1.7	2.5	1.6	2.6	4	25.8	20.4	5	7.8	5
Kelishadi et al. 2006 [54]	Iran	4811	2248	2563	6–18	–	678 (14)	–	–	–	–	–	23	72	38	4	7	6
Esmaillzadeh et al. 2006 [55]	Iran	3036	1413 (10.3)	1623 (9.9)	10–19	–	307 (10.1)	–	3.9	6.2	4.8	5.3	10	42.8	37.5	0.6	23.8	6
Rodríguez-Morán et al. 2004 [56]	Mexico	965	499 (4.6)	466 (8.6)	10–18	–	63 (6.5)	–	–	–	2.4	4.1	27.7	20.8	9.5	7.7	7.1	6

### Prevalence of MetS and components among overweight and obese children and adolescents

The pooled prevalence of MetS was estimated based on the three diagnostic methods (IDF, ATP III and de Ferranti). A total of 14 articles [35, 47, 55, 58, 63, 69, 72, 77, 79, 87–89, 92, 95] were eligible to compute the pooled prevalence of MetS in the IDF criteria. Accordingly, 24.1% (95% CI 16.90, 31.29, I^2^ = 96.6%) of the study subjects were found to have MetS. Abdominal obesity was the most common (60.9%) component of MetS, whereas high FG level was the least (10.3%) component. According to the modified ATP III, the pooled prevalence of MetS was 36.51% (95% CI − 1.76, 74.78, I^2^ = 99.8%). It was computed using eight articles [50, 57, 63, 71, 77, 84, 89, 104]. Two thirds (67.2%) of the children and adolescents were found to have abdominal obesity, but very few (3.4%) of them had high FG level. Besides, only two articles [89, 91] were eligible to estimate the pooled prevalence of MetS (56.32%, 95% CI 22.34, 90.29, I^2^ = 94.4%) among overweight and/or obese children and adolescents in accordance with de Ferranti criteria. Similarly, abdominal obesity and high FG level were the most (91.2%) and least (7.75%) components of MetS in the de Ferranti diagnostic criteria.

**Fig. 2:**
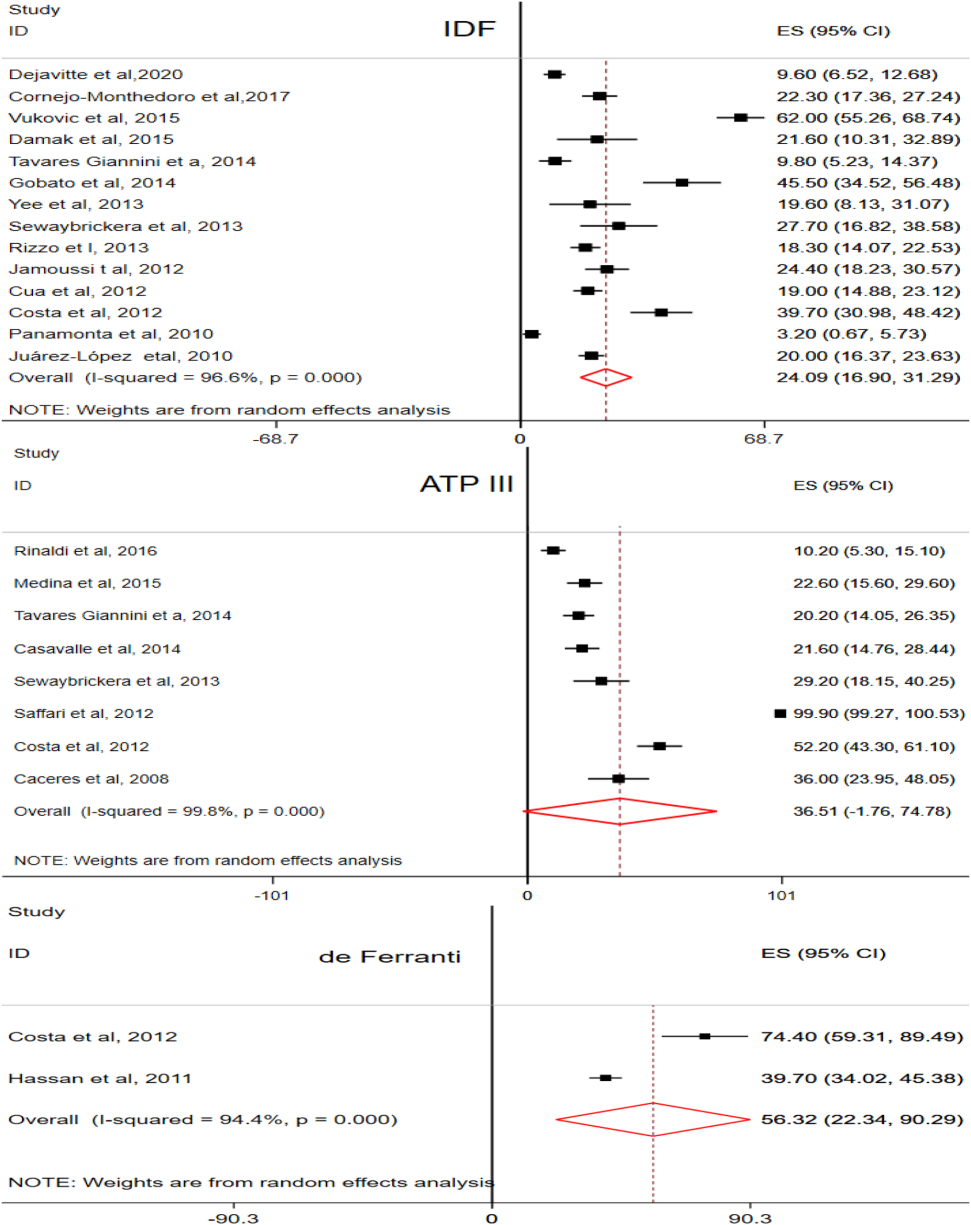
The pooled prevalence of MetS in overweight and obese children and adolescents

The pooled prevalence of MetS was also computed based on gender. The prevalence of MetS was relatively higher in males (26.63%) than females (24.05%) in the IDF method. Likewise, males (33.37%) were highly affected by MetS than females (31.4%) according to the modified ATP III diagnostic criteria (Fig. 2 & Table 4).

**Table 4:** Pooled prevalence of MetS & components in overweight & Obese children and adolescents

Variables	Characteristics	# of studies	Pooled prevalence, (95% CI)	Heterogeneity (I^2^(%), P-value))	Model
Diagnostic Criteria	IDF	14	24.09 (16.90, 31.29)	96.6, P ≤ 0.001	REM
	ATP III	8	36.51 (− 1.76, 74.78)	99.8, P ≤ 0.001	REM
	de Ferranti	2	56.32 (22.34,90.29)	94.4, P ≤ 0.001	REM
Components of MetS (IDF)	Abdominal Obesity	12	60.90 (46.63,75.16)	99.7, P ≤ 0.001	REM
	Low HDL-C	13	34.83 (23.8, 46.48)	98.0, P ≤ 0.001	REM
	High TG	12	18.59 (13.21,23.98)	93.0, P ≤ 0.001	REM
	High FG	13	10.27 (6.67,13.87)	95.9, P ≤ 0.001	REM
	Elevated BP	13	23.88 (17.29, 30.47)	99.8, P ≤ 0.001	REM
Components of MetS (ATPIII)	Abdominal Obesity	8	67.20 (49.45,84.95)	98.9, P ≤ 0.001	REM
	Low HDL-C	8	42.48 (33.45, 51.51)	99.8, P ≤ 0.001	REM
	High TG	8	38.85 (27.61, 50.10	92.9, P ≤ 0.001	REM
	High FG	7	3.39 (1.05,5.74)	81.4, P ≤ 0.001	REM
	Elevated BP	8	29.56 (15.03, 44.8)	96.9, P ≤ 0.001	REM
Components of MetS (de Ferranti)	Abdominal Obesity	2	91.20 (80.42, 101.98)	95.6, P ≤ 0.001	REM
	Low HDL-C	2	62.29 (2.91, 121.68)	99.7, P ≤ 0.001	REM
	High TG	2	42.40 (38.39, 46.40)	0.00, P = 0.632	FEM
	High FG	2	7.75 (− 4.20, 19.71)	97.3, P ≤ 0.001	REM
	Elevated BP	2	53.04 (8.25, 97.82)	99.1, P ≤ 0.001	REM
Gender (IDF)	Male	10	26.63 (23.95, 29.31)	99.3, P ≤ 0.001	REM
	Female	10	24.05 (16.65, 31.45)	90.7, P ≤ 0.001	REM
Gender (ATPIII)	Male	5	33.37 (19.68, 47.06)	99.5, P ≤ 0.001	REM
	Female	5	31.40 (15.43, 47.36)	99.8, P ≤ 0.001	REM

REM, random effect model; FEM, fixed effect model

### Prevalence of MetS & components in the general population of children & adolescents

The pooled prevalence of MetS was estimated in LMICs using the IDF, ATP III and de Ferranti diagnostic methods. A total of 30 [33, 36–38, 40–44, 46, 48, 51, 52, 54, 60, 62, 68, 70, 73–75, 78, 80, 81, 83, 85, 90, 94, 98, 102], 33 [34, 39, 42, 43, 51, 53, 56, 59–62, 65–67, 73–76, 82, 85, 86, 93, 96–102, 105–108], and 8 [42, 45, 49, 51, 64, 75, 78, 103] articles were eligible to compute the pooled estimates in the IDF, ATP III and de Ferranti diagnostic criteria, respectively.

According to the IDF criteria, the pooled prevalence of MetS among the general population of children and adolescents was 3.98% (95% CI 3.35, 4.61, I^2^ = 97.8%). The pooled estimate in males (3.46%; 95% CI 2.69, 4.23, I^2^ = 97.6%) was relatively higher than females (2.99%; 95% CI 2.34, 3.65, I^2^ = 95.6%). From the components, low HDL-C level was the commonest (27.93%) and high FG (7.78%) was the infrequent one.

Similarly, 6.71% (95% CI 5.51, 7.91, I^2^ = 97.6%) study subjects were found to have MetS in the ATP III criteria. MetS among males (6.24%; 95% CI 4.89, 7.59, I^2^ = 93.9%) and females (6.51%; 95% CI 4.99, 8.03, I^2^ = 95.8%) was nearly the same. Low HDL-C was seen in one third (31.3%; 95% CI 23.89, 38.72, I^2^ = 99.7%) of study subjects and high FG in 6.1% (95% CI 5.02, 7.15, I^2^ = 98.7%) of study subjects.

**Fig. 3:**
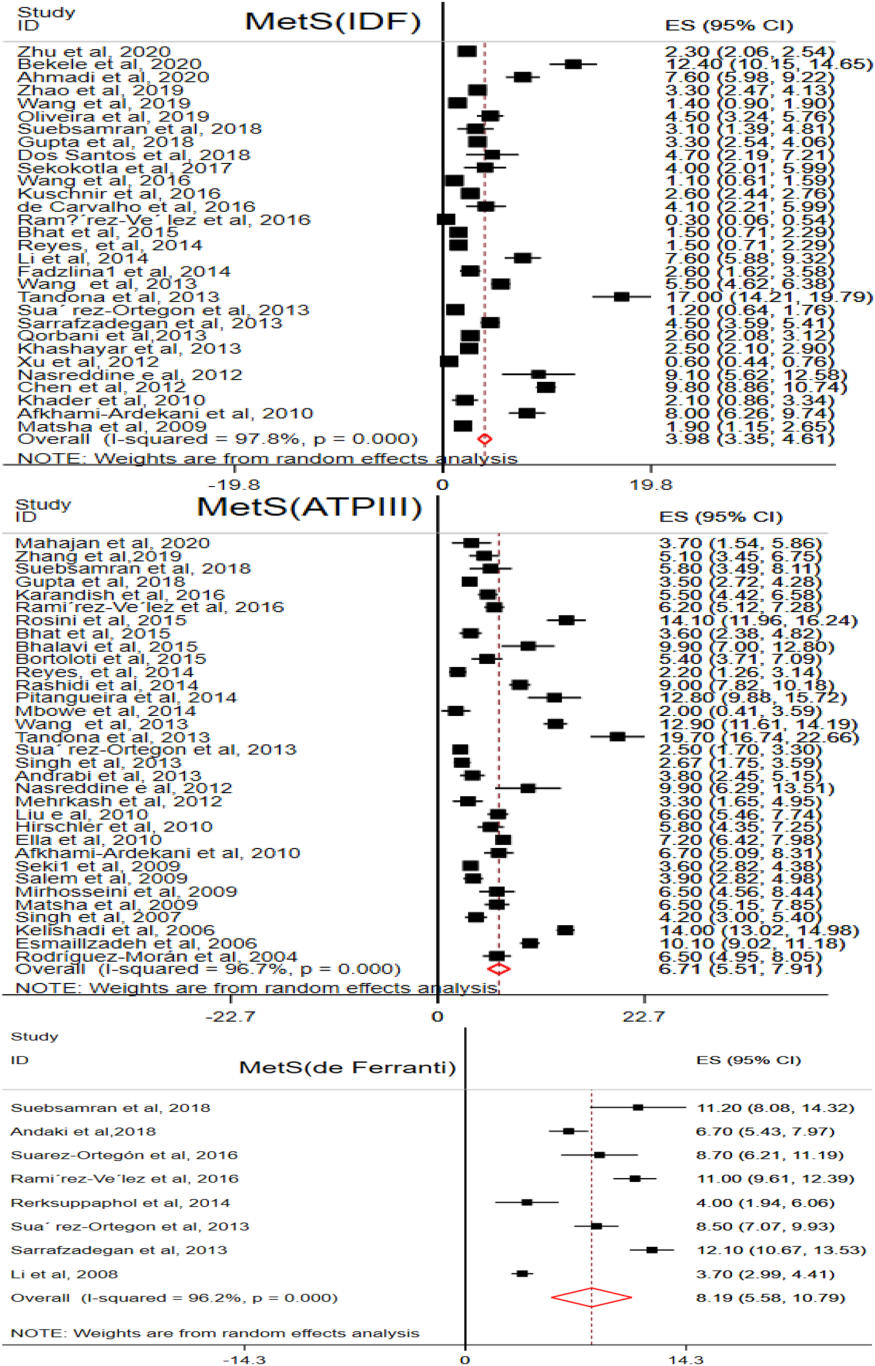
Metabolic Syndrome among children and adolescents in the general population

Besides, the pooled prevalence of MetS in children and adolescents with de Ferranti diagnostic method was 8.19% (95% CI 5.58, 10.79, I^2^ = 96.2%) with similar prevalence in males (8.78%; 95% CI 5.45, 12.12, I^2^ = 94.3%) and females (8.51%; 95% CI 5.21, 11.75, I^2^ = 93.7%). The pooled estimate of low HDL-C was 45.83% (95% CI 34.53, 57.14, I^2^ = 99.1%), the highest, and only 2.12% (95% CI 1.15, 3.08, I^2^ = 94.7%) of the population had a high FG level (Fig. 3 & Table 5).

**Table 5:** The pooled prevalence of MetS and components in the general population

Variables	Characteristics	# included articles	Pooled prevalence (95%, CI)	Heterogeneity (I^2^ (%), P-value)	Model
Diagnostic Criteria	IDF	30	3.98 (3.35,4.61)	97.8, P ≤ 0.001	REM
	ATP III	33	6.71 (5.51, 7.91)	96.7, P ≤ 0.001	REM
	de F	8	8.19 (5.58, 10.79)	96.2, P ≤ 0.001	REM
Gender distribution of MetS (IDF)	Male	20	3.46 (2.69, 4.23)	96.7, P ≤ 0.001	REM
	Female	20	2.99 (2.34, 3.65)	95.6, P ≤ 0.001	REM
Gender distribution of MetS (ATPIII)	Male	24	6.24 (4.89, 7.59)	93.9, P < 0.001	REM
	Female	26	6.51(4.99, 8.03)	95.8, P ≤ 0.001	REM
Gender distribution of MetS (deF.)	Male	7	8.78 (5.45, 12.12)	94.3, P ≤ 0.001	REM
	Female	7	8.51 (5.21, 11.75)	93.7, P ≤ 0.001	REM
Study Population (IDF)	Overweight & Obese	11	1.48 (0.94, 2.01)	87.8, P ≤ 0.001	REM
	Others^a^	12	0.58 (0.33, 0.82)	93.2, P ≤ 0.001	REM
Study Population (ATP III)	Overweight & Obese	18	4.66 (3.49, 5.83)	95.7, P ≤ 0.001	REM
	Others	19	2.31 (1.53, 2.72)	95.7, P ≤ 0.001	REM
Study Population (de F.)	Overweight & Obese	4	3.95 (1.82, 6.08)	93.3, P ≤ 0.001	REM
	Others^a^	4	3.20 (0.78, 5.62)	96.4, P ≤ 0.001	REM
Components MetS (IDF)	Abdominal obesity	25	18.85 (16.39, 21.31)	98.9, P ≤ 0.001	REM
	Low HDL-C	25	27.93 (21.91, 33.96)	99.8, P ≤ 0.001	REM
	High TG	26	11.09 (9.13, 13.05)	99.3, P ≤ 0.001	REM
	High FG	26	7.78 (6.40, 9.15)	99.0, P ≤ 0.001	REM
	Elevated BP	25	8.76 (7.22, 10.29)	99.1, P ≤ 0.001	REM
Components MetS (ATP III)	Abdominal obesity	18	4.66 (3.49, 5.83)	95.7, P ≤ 0.001	REM
	Low HDL-C	28	31.30 (23.89, 38.72)	99.7, P ≤ 0.001	REM
	High TG	28	21.05 (16.63,25.48)	99.4, P ≤ 0.001	REM
	High FG	28	6.08 (5.02, 7.15)	98.7, P ≤ 0.001	REM
	Elevated BP	27	12.27 (9.39, 15.16)	99.1, P ≤ 0.001	REM
Components MetS (de F.)	Abdominal obesity	7	22.65 (14.01, 31.39)	99.3, P ≤ 0.001	REM
	Low HDL-C	6	45.83 (34.53, 57.14)	99.1 P ≤ 0.001	REM
	High TG	7	17.4 (12.24, 21.84)	97.3 P ≤ 0.001	REM
	High FG	7	2.12 (1.15, 3.08)	94.7, P ≤ 0.001	REM
	Elevated BP	7	12.86 (7.11, 18.61)	98.7, P ≤ 0.001	REM

^a^Others: underweight and normal weight, REM, Random Effect Model; de F., de Ferranti

### Subgroup analysis of the pooled prevalence of MetS in the general population

**Fig. 4:**
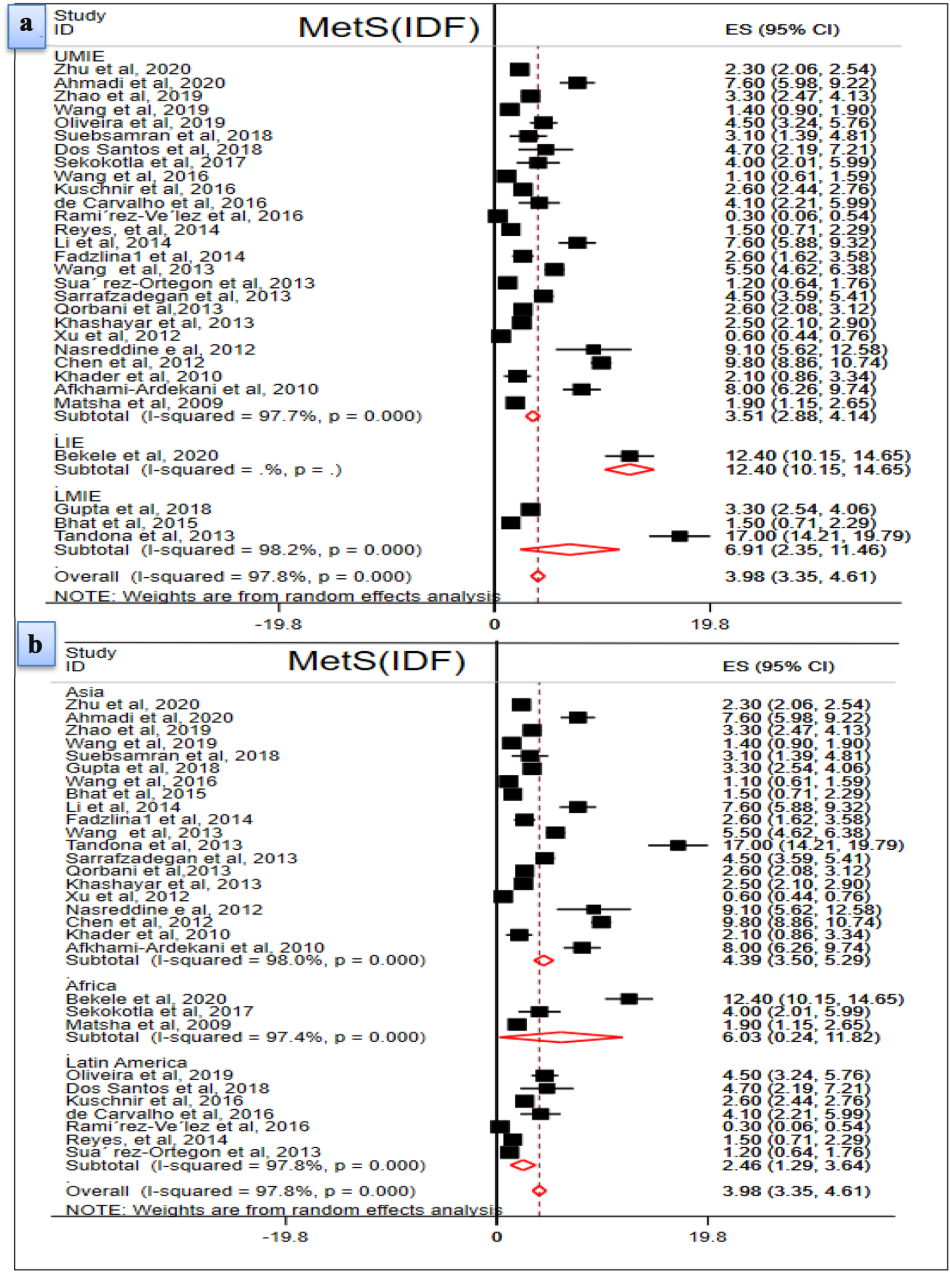
Pooled prevalence of MetS (**a** Subgroup analysis using income level; **b** Subgroup analysis based on continent)

The subgroup analyses were performed for the two diagnostic methods (IDF and ATP III) using the two parameters (income level and continent). In the IDF diagnostic method, the pooled estimate of MetS in LIE, LMIE and UMIE countries were estimated. The prevalence of MetS in LIEs (12.4%, 95% CI 10.5, 14.65) was computed from one study. Likewise, the pooled estimates of MetS in LMIE (6.91%; 95% CI 2.35, 11.46, I^2^ = 98.2%) and UMIE (3.51%; 2.88, 4.14, I^2^ = 97.7%) countries were computed from three and 26 articles, respectively. Regarding the continent where the original studies were conducted, only three articles were from Africa, seven articles from Latin America and the majorities (20) articles were from Asia. The pooled prevalence of MetS in Africa, Asia and Latin America were 6.03% (95% CI 0.24, 11.28, I^2^ = 94.7%), 4.39% (95% CI 3.50, 5.29, I^2^ = 98%), and 2.46% (95% CI 1.29, 3.64, I^2^ = 97.8%), respectively (Fig. 4).

**Fig. 5:**
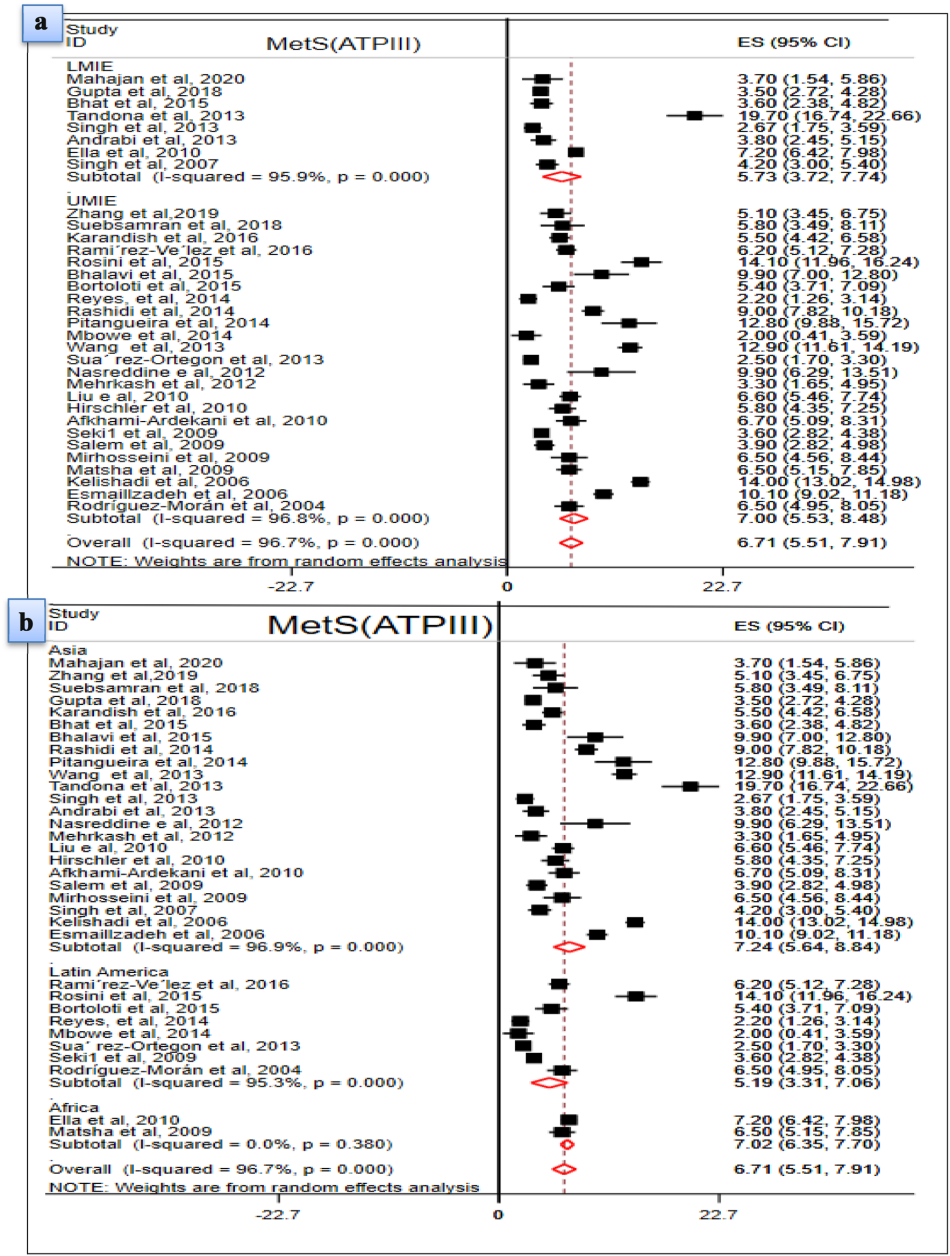
Pooled prevalence of MetS (**a** Subgroup analysis using income level; **b** Subgroup analysis using continent)

According to the ATP III diagnostic method, the pooled prevalence of MetS in countries classified under LMIE and UMIE was estimated from eight and 25 eligible articles, respectively. Accordingly, 5.73% (95% CI 3.72, 7.74, I^2^ = 95.9%) of the study subjects in LMIEs and 7% (95% CI 5.53, 8.48, I^2^ = 96.8%) in UMIE countries were found to have MetS. The pooled prevalence of MetS in Africa, Latin America and Asia was computed from two, eight and 23 articles, respectively. Thus, 6.71% (95% CI 5.51, 7.91, I^2^ = 0.00%) in Africa, 5.19% (95% CI 3.31, 7.05, I^2^ = 95.3%) in Latin America and 7.24% (95% CI 5.64, 8.84%, I^2^ = 96.9%) in Asia had MetS (Fig. 5).

### Publication bias and sensitivity analysis

**Fig. 6:**
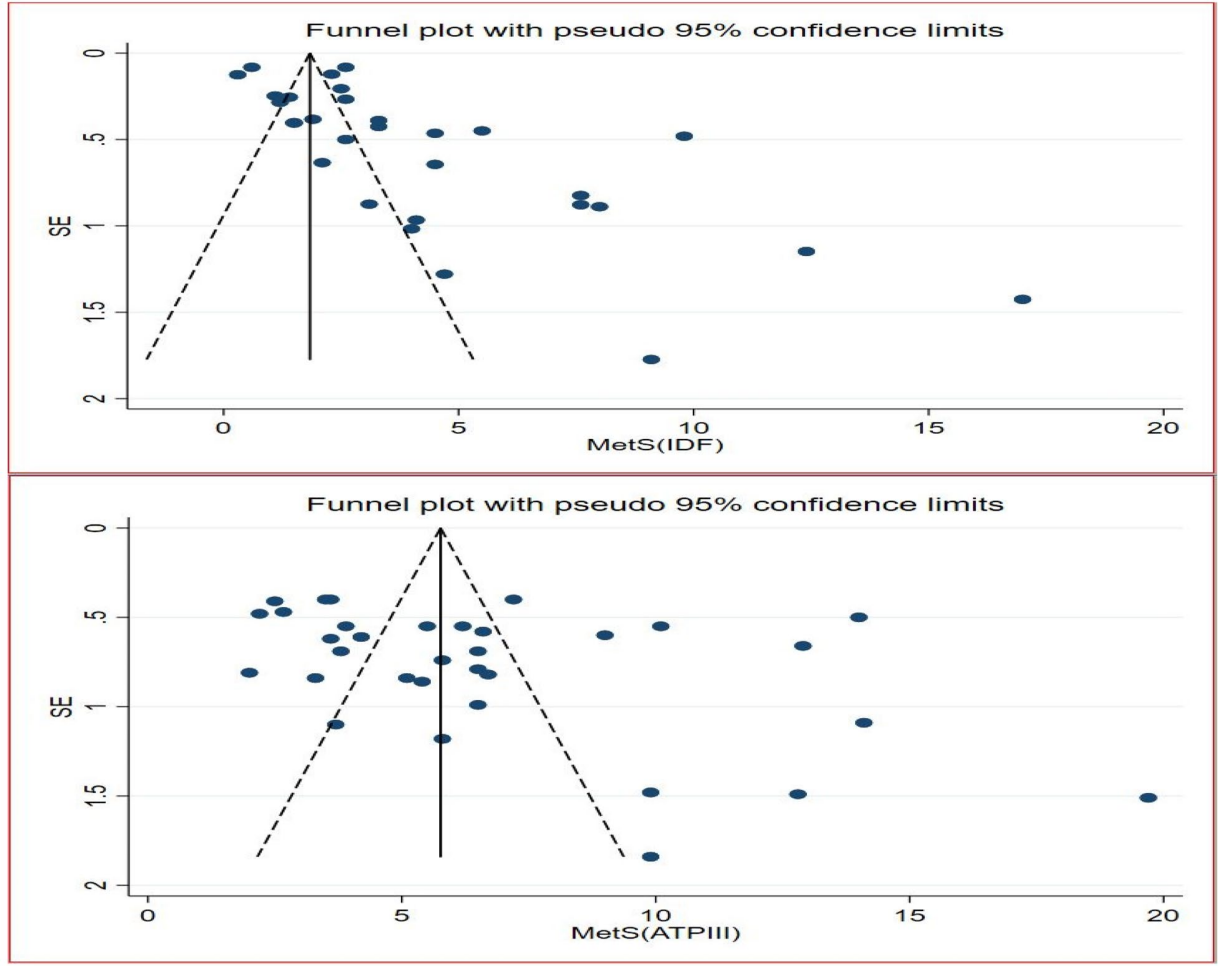
Funnel plot for the two diagnostic methods (IDF & ATP III)

**Fig. 7:**
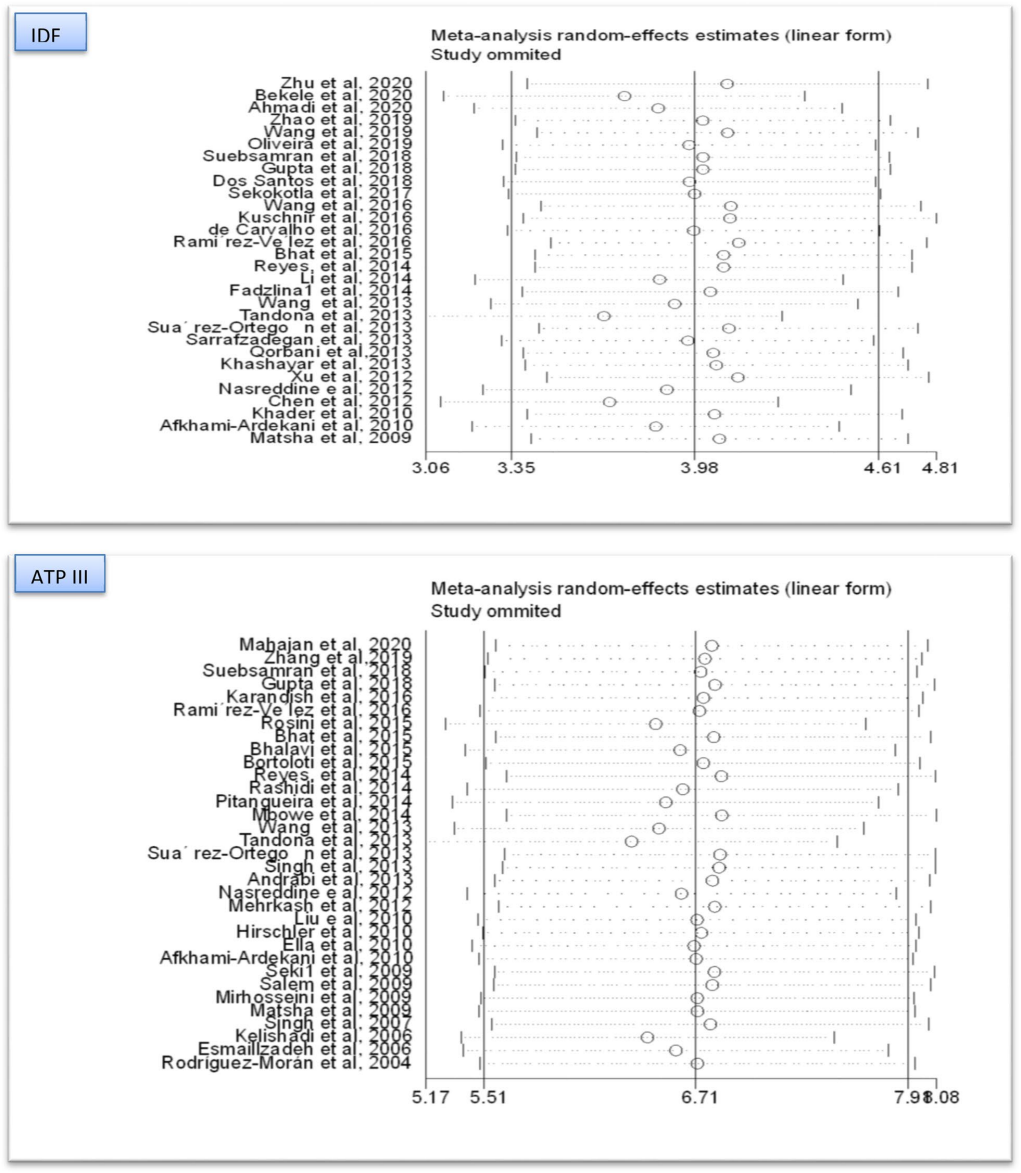
Sensitivity analysis for two diagnostic methods (IDF & ATPIII)

Due to the presence of high heterogeneity among the included articles, the possible sources of variation were further explained. Thus, the funnel plots for both IDF and ATP III diagnostic criteria were presented (Fig. 6). The asymmetry of plots was objectively verified by Egger’s regression test and there was publication bias among the articles included in computing the pooled prevalence of MetS in the IDF criteria (P = 0.001), whereas the Egger’s regression test revealed that there was no publication bias in the pooled estimate of ATP III diagnostic criteria (P = 0.063). Moreover, sensitivity analysis was computed for both diagnostic methods. This was done to evaluate if the pooled estimates were altered by the exclusion of any single study. However, none of the studies had significant effects in the pooled estimates (Fig. 7).

**Fig. 8:**
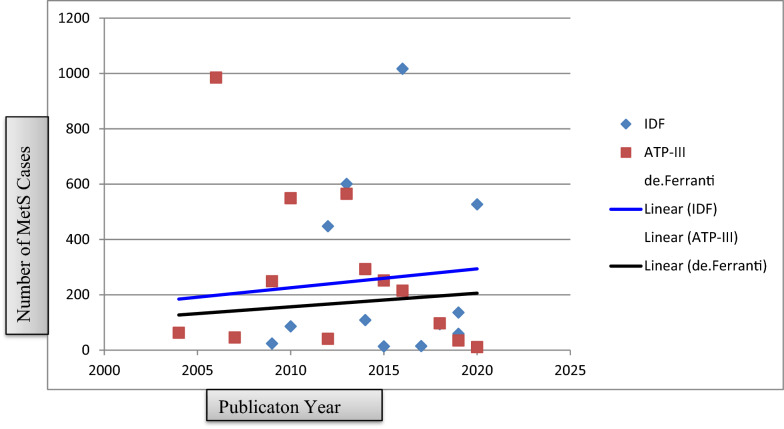
Time trend of metabolic syndrome among children and adolescents in LMICs from 2004 to 2020

Finally, the prevalence of MetS in LMICs among the general population children and adolescents was plotted in linear graph using the number of cases with publication year (2004 to 2020). The graph depicted that there is an increasing trend in the two diagnostic methods (IDF & de Ferranti) and the reverse was true in ATP III criteria (Fig. 8).

## Discussion

To the authors’ knowledge, this is the first comprehensive systematic review and meta-analysis, determining the prevalence of metabolic syndrome among children and adolescents in LMICs. In this study, the pooled prevalence of MetS was computed using three diagnostic methods: IDF, ATP III & de Ferranti. Seventy six articles with 142,142 participants were included in this meta-analysis. Of the total studies, 56 were conducted among the general population of study subjects, and 20 were conducted among overweight and/or obese children and adolescents.

The current meta-analysis revealed that the prevalence of MetS among overweight and obese population is remarkably higher than the general population of children and adolescents. The pooled prevalence of MetS in the overweight and obese children and adolescents is as follows: IDF: 24.1%, ATP III: 36.5% and de Ferranti: 56.32%. Whereas, it is 3.98%, 6.71% and 8.19% with the IDF, ATP III and de Ferranti diagnostic methods, respectively in the general population. The prevalence in the general population is comparable with a review done in Iran, where the prevalence of MetS was 0–8%, 3–16%, and 0–22% in the IDF, ATP III and de Ferranti criteria, respectively [18]. However, the current prevalence among the overweight and obese population is considerably higher than the Iranian review findings [18]. The possible explanation for this variation could be due to the fact that overweight and obese children are at greater risk of developing metabolic syndrome as compared to children with normal weight [20]. The present findings are also in line with the previous review findings which reported that the prevalence of MetS in the pediatric population ranged from 1.2–22.6% [109] to 0–19.2% [15] irrespective of the specific diagnostic methods. The median prevalence of MetS in the world was 3.3% in 2007 to 2009, which is lower than the all pooled estimates in this meta-analysis [15]. The prevalence of MetS is also considerably higher than a meta-analysis findings done in Chinese children and adolescents, where 1.8% (IDF) and 2.6% (ATP III) were found to have MetS [110]. This implies that MetS is increasing throughout the world, including in LMICs and it is supported by the previous reviews [14, 111, 112].

Regarding gender based distribution; the prevalence of MetS in males is relatively higher than in females in most of the diagnostic methods. The prevalence among overweight and obese males is 26.63% (IDF) and 33.37% (ATP III), and it is 24.05% (IDF) and 31.4% (ATPIII) among females. Similarly, the prevalence of MetS among males (3.46%) in the general population is higher as compared to females (2.99%) with IDF criteria. However, the pooled prevalence of MetS among males and females in the general population of children and adolescents is approximately similar in the two diagnostic methods (ATPIII & de Ferranti). The prevalence in males is 6.24% (ATPIII) and 8.78% (de Ferranti); and it is 6.51% (ATPIII) and 8.51% (de Ferranti) among females. In general, males are at greater risk to have MetS than females. This finding is supported by most of the original studies included in this meta-analysis and the other meta-analysis done in china [110]. The possible justification for gender disparities could be related to higher prevalence of obesity in males than females. This could be further explained by the fact that males usually consume excessive energy due to self and family perceived underweight and underestimation of their weight. On the contrary, female adolescents control their weight through diet and physical activity due to self-perceived overweight [113]. But, further exploration is needed with experimental studies.

The pooled prevalence of the components of MetS was also computed in each of the diagnostic methods and considerable numbers of study subjects were found to have each of the five components. Abdominal obesity is found to be the commonest component of MetS in overweight and obese children, with a pooled prevalence of 60.9% (IDF), 67.2% (ATP III), and 91.2% (de Ferranti). In contrary, a high FG level was the most infrequent component of MetS with a pooled prevalence of 10.3% (IDF), 3.4% (ATP III), and 7.75% (de Ferranti). Besides, the pooled prevalence of low HDL-C is the most prevalent component of MetS among the general population. It was found in 27.93% (IDF), 31.3% (ATP III) and 45.83% (de Ferranti) of the study population. But, high FG is the least component in IDF (7.78%) and de Ferranti (2.12%) criteria. Likewise, abdominal obesity is the least (4.46%) component in the ATP III criteria. All the other component of MetS in overweight and obese children is considerably higher as compared to the pooled prevalence in the general population. The possible elucidation could be due to a multitude of factors like consumption of unhealthy diets (Western type of diets), diets low in fruit, vegetables, fruits and grains [114, 115].

Moreover, children and adolescents from countries with UMIEs are found to have a lower risk of developing MetS than children from countries classified under LIE and LMIE in the IDF criteria. The pooled prevalence of MetS in LIE, LMIE, and UMIE countries is 12.4%, 6.91% and 12.4%, respectively. However, the prevalence of MetS in LMIE (5.3%) is relatively lower than UMIE (7%) countries in ATP III diagnostic criteria. These findings remind that MetS is an emerging crisis in children and adolescent without geographical boundary. This could be primarily associated with the nutrition transition in developing countries [116].

In addition, MetS was calculated based on the continent where the original studies were conducted. In the IDF criteria, the pooled prevalence in Africa (6.03%) is relatively higher than in Asia (4.39%) and Latin America (2.46%). Whereas, the pooled prevalence of MetS in Africa (7.02%) and Asia (7.24%) are nearly the same in the general population and higher than the prevalence in Latin America (5.19%) in the ATP III diagnostic criteria. The rising burden of MetS in the poor continents like Africa is corroborated by the fact that the universal increment of obesity, the main predictor of MetS in the world, including the poorest LMICs [117, 118]. Finally, the number of cases was plotted against the publication year. It was pinpointed in the line graph that there is an increasing trend in the two diagnostic methods (IDF and de Ferranti), but the trend decreases from 2004 to 2020 in the ATP III diagnostic method. This may be attributed to differences in the sample size. The other possible rational could be related to variation in the year of publication of the original studies.

The findings of this study will have a vital implication for program planners and policy makers to design preventive strategies accordingly. These findings will have several implications for the poorest LMICs, where nutrition transition and the triple burden of malnutrition are prevailing in recent years. However, the issue of which diagnostic method is the best remains unresolved and this could affect the final conclusion. The other limitation of this study was some articles written other than the English language and articles with the difficulty of accessing the full texts were excluded. We excluded articles which are not written in English because it is difficult to identify the real estimates of MetS and to assess the qualities of studies. Studies conducted among different population, such as studies among children with type 1 diabetic were excluded to avoid overestimation of MetS. This could affect the pooled estimates of MetS and components.

## Conclusion

In conclusion, this study illustrates that MetS among children and adolescents is an emerging public health challenge in LMICs where the prevalence of obesity is on the move. The prevalence is significantly higher among the overweight and obese population. The burden is also rising in low income countries found in Asia and Africa. Metabolic syndrome was diagnosed in underweight, normal weight, overweight and obese children and adolescents, revealing the triple burden of malnutrition in these countries. Thus, additional studies need to be conducted to identify all possible factors. Preventive strategies like community based and school based interventions on lifestyle modifications may avert MetS in LMICs. Specifically, promoting healthy eating behaviors and physical activities as well as avoiding consumption of fructose based drinks may primarily decrease the burden.

## Supplementary information

**Additional file 1.** Extraction checklist.

**Additional file 2.** Critical appraisal of the included studies.

**Additional file 3.** List of excluded studies.

## Data Availability

The data that support the review findings of this study are included in the manuscript and with Additional file 1, 2, 3.

## Abbreviations

HDL-C: High density lipoprotein-cholesterol
Mets: Metabolic syndrome
IDF: International Diabetic Federation
TG: Triglyceride
BP: Blood Pressure
FG: Fasting glucose
WHO: World Health Organization
BMI: Body mass index
ATP III: National Cholesterol Education Program (NCEP) Adult Treatment Panel-III
FG: Fasting glucose
WC: Waist circumference
LMICs: Low and Middle Income Countries
NCD: Noncommunicable diseases
LIE: Low Income Economies
LMIE: Lower Middle Income Countries,
UMIE: Upper Middle Income
JBI: Juana Brigg’s Institute
