# Supplementary material for: Metabolic syndrome among children and adolescents in low and middle income countries: a systematic review and meta-analysis
Source: Diabetol Metab Syndr. 2020 Oct 27;12:93. doi: 10.1186/s13098-020-00601-8 (PMC7590497; doi:10.1186/s13098-020-00601-8)
Supplement: Supplementary file 1 — Additional file 1. Extraction checklist. [file 13098_2020_601_MOESM1_ESM.docx]

Data extraction format for studies included to compute the prevalence of metabolic syndrome in low and middle income countries

| Author, year | Country | Sample size | M/F | Age | MetS with Diagnostic method N(%) | | | S. Design | Population | | Gender | | Components of Mets | | | | |
| --- | --- | --- | --- | --- | --- | --- | --- | --- | --- | --- | --- | --- | --- | --- | --- | --- | --- |
|  |  |  |  |  | IDF | ATP-III | de.F. |  | Non-obese | Overwt./Obese | M, N(%) | F, N(%) | Ab. obesity | Low HDL | High TGL | High FG | High BP |
| Zhu et al, 2020 | China | 15045 | 7711/7334 | 7-18 | 346(2.3) | - | - | CS | - | - | 216(1.4) | 130(0.9) | 3282(21.8) | 2163(14.4) | 826(5.5) | 454(3) | 559(3.7) |
| Mahajan et al, 2020 | India | 296 | 128/168 | 14-19 | - | 11(3.7) | - | CS | - | - | 5 (1.7) | 6 (2.1) | 29 (9.8) | 192 (64.9) | 19 (6.4) | 1 (0.3) | 50 (16.9) |
| Dejavitte et al,2020 | Brazil | 354 | 142/212 | 10-19 | 34(9.6) | - | - | CS | - | 34(9.6) | 22 (6.2) | 12 (3.4) | 274(77.4) | 175(49.4) | 20(5.6) | 53(15) | 4 (1.1) |
| Bekele et al,2020 | Ethiopia | 824 | 403/421 | 13-19 | 102(12.4) | - | - | CS | 52 (6.3) | 50(6.1) | 41 (5) | 61(7.4) | 265 (32.2) | 170(20.6) | 216 (26.2) | 476(57.8) | 70(8.5) |
| Ahmadi et al,2020 | Iran | 1035 | 456/579 | 6-18 | 79(7.6) | - | - | CS | - | - | 45(4.3) | 34 (3.3) | 277(27.8) | 438(56.2) | 58 (7.4) | 71(9.1) | 76 (8) |
| Zhao et al, 2019 | China | 1766 | 871/895 | 10-15 | 3.3 (59) | - | - | CS | 2 (0.1) | 57 (3.2) | 35 (2) | 24 (1.3) | 538 (30) | 78 (4) | 171 (10) | 187 (11) | 121 (7) |
| Zhang et al,2019 | China | 683 | 366/317 | 8–15 | - | 35(5.1) | - | CS | 1 (0.1) | 34 (5) | 24(3.5) | 11(1.6) |  | - | - | - | - |
| Wang et al,2019 | China& Spain | 2126 | 1011/1115 | 10-15 | 30(1.4) | - | - | CS | - | - | - | - | 114(16.7) | 336(15.8) | 116(5.5) | 88(4.1) | 267 (12.6) |
| Oliveira et al,2019 | Brazil | 1035 | 470/565 | 12-20 | 47 (4.5) | - | - | CS | 35(3.4) | 12(1.1) | 25(2.4) | 22(2.1) | 154 (14.9) | 273 (26.4) | 43 (4.2) | 46 (4.4) | 93 (9.0) |
| Suebsamran et al,2018 | Thailand | 393 | 152/241 | 13-16 | 12 (3.1) | 23(5.8) | 44(11.2) | CS | 1(0.3) | 11(2.8) | 9(2.3) | 3(0.8) | 64 (15.6) | 102 (25.6) | 13 (3.3) | 3 (0.8) | 19 (4.6) |
|  |  |  |  |  |  |  |  | CS | 4(1) | 19(4.8) | 16 (4.1) | 7 (1.7) | 64 (15.6) | 113 (28.4) | 56 (13.7) | 1 (0.2) | 48 (11.7) |
|  |  |  |  |  |  |  |  | CS | 12(3.1) | 32(8.1) | 24 (6.1) | 20 (5.1) | 140 (34.6) | 204 (51.3) | 73 (17.8) | 1 (0.2) | 48 (11.7) |
| Gupta et al, 2018 | India | 2100 | 1149/951 | 10-16 | 69(3.3) | 74(3.5) | - | CS | - | - | 51(2.4) | 18(0.9) | 168 (8.0) | 354 (16.9) | 193 (9.2) | 283 (13.5) | 159 (7.6) |
| Dos Santos et al, 2018 | Brazil | 274 | 88/186 | 12-18 | 13(4.7) | - | - | CS | - | - | 5(1.8) | 8(2.9) | 42 (15.3) | 69 (25.2) | 18 (6.6) | 14 (5.1) | 24 (8.8) |
| Andaki et al,2018 | Brazil | 1480 | 707/773 | 6-10 | - | - | 99(6.7) | CS | - | - | 89(6) | 10(07) | 407(27.5) | 636(43) | 159 (10.7) | 11(0.7) | 159 (10.7) |
| Sekokotla et al, 2017 | S.Africa | 371 | 116/255 | 13-18 | 15(4) | - | - | CS | - | - | 7(1.9) | 8(2.1) | 111(30) | 107(28.8) | 32(8.6) | 17(4.6) | 121 (32.6) |
| Cornejo-Monthedoro et al,2017 | Peru | 273 | 143/130 | 10-15 | 61(22.3) | - | - | CS | - | 61(22.3) | 28(10.3) | 33(12) | 223(81.7) | 174(63.7) | 81(29.7) | 16(5.9) | 14(5.1) |
| Wang et al, 2016 | China | 1770 | 857/913 | 7-17 | 19(1·1) | - | - | CS | - | - | 12(0.68) | 7(0.42) | 210(11.9) | 206(11.6) | 98(5.5) | 28(1.6) | 14(0.8) |
| Suarez-Ortegón et al, 2016 | Colombia | 494 | 256/238 | 5-9 | - | - | 43(8.7) | CS | - | - | 22(4.5) | 21(4.2) | 163(33) | 494 (47.6) | 101 (20.4) | 20(4) | 13(2.6) |
| Rinaldi1 et al, 2016 | Brazil | 147 | 71/76 | 6-10 | - | 15(10.2 | - | CS | - | 15(10.2 | 9(6.1) | 6(4.1) | 70(47.6) | 36(24.5) | 35(23.8) | 1(0.8) | 17(11.6) |
| Kuschnir et al, 2016 | Brazil | 37504 | 15006 / 22498 | 12-17 | 975(2.6) | - | - | CS | - | - | 435(1.2) | 540(1.4) | 4725(12.6) | 12189 (32.7) | 1725 (4.6) | 1538 (4.1) | 3075 (8.2) |
| Karandish et al, 2016 | Iran | 1749 | 886/863 | 10-16 | - | 96(5.5) | - | CS | - | - | 71(4.1) | 25(1.4) | 163(9.2) | 443(25) | 558 (31.2) | 302 (17) | 405 (22.8) |
| de Carvalho et al, 2016 | Brazil | 421 | 170/251 | 9-19 | 17(4.1) | - | - | CS | - | - | - | - | 36(8.6) | 110(26.1) | 88(20.9) | 2(0.5) | 50(11.9) |
| Vukovic et al, 2015 | Serbia | 254 (199) | 84/115 | 4-19 | 62 (31.2) | - | - | CS | - | 62(31.2) | 28 (14.1) | 34 (17.1) | 23(9.1) | 115(45.3) | 40(15.7) | 11(4.3) | 88(34.6) |
| Rosini et al, 2015 | Brazil | 1011 | 481/530 | 6-14 | - | 143  (14.1) | - | CS | 31(3) | 112(11.1) | 63(6.2) | 80(7.9) | 307(30.4) | 380(37.6) | 264  (26.1) | 117 (11.6) | 137  (13.6) |
| Medina et al, 2015 | Mexico | 137 | 67/70 | 6-12 | - | 31  (22.6) | - | CS | - | 31(22.6) | 19 (13.9) | 12(8.7) | 78(56.9) | 47(34.3) | 63(46) | 1(0.73) | 29(21.1) |
| Bhat et al, 2015 | India | 899 | 311/588 | 10-18 | 14(1.5) | 32(3.6) | - | CS | 15(1.7) | 17(1.9) | 12(1.4) | 20(2.2) | 33(3.7) | 153(17) | 279(31) | 88(9.8) | 36(4)_  ATP |
| Bhalavi et al, 2015 | India | 405 | 182/223 | 10-19 | - | 40(9.9) | - | CS | 40(9.9) | - | 14(3.5) | 26(6.4) | 9(2.2) | 236(58.3) | 113  (27.9) | 56  (13.8) | 91(22.4) |
| Reyes, et al, 2014 | Venezuela | 916 | 450/466 | 9-18 | 14 (1.5) | 20 (2.2) | - | CS | - | - | 14(1.5) | 6(0.7) | 93 (10.2) | 79 (8.6) | 96 (10.5) | 33(3.6) | 79 (8.7) |
|  |  |  |  |  |  |  |  |  |  |  |  |  | 87 (9.5) | 288 (31.4) | 69 (7.5) | 33(3.6) | 6 (0.7) |
| Tavares Giannini et a, 2014 | Brazil | 163 | 52/111 | 10-18 | 16(9.8) | 33  (20.2) | - | CS | - | 33(20.2) | - | - | 140(85.9) | 69(42.3) | 48(29.4) | - | 22(13.5) |
|  |  |  |  |  |  |  |  |  |  |  |  |  | 115(70.5) | 39(23.9) | 14(8.6) | 3(1.8) | 30(18.4) |
| Rerksuppaphol et al, 2014 | Thailand | 348 | 189/159 | - | - | - | 14(4)- | CS | 2(0.6) | 12(3.4) | 7(2) | 7(2) | 103(29.6) | - | 44(12.6) | 31(8.9) | 64(18.4) |
| Rashidi et al, 2014 | Iran | 2246 | 1113/1133 | 10–19 | - | 203(9) | - | CS | 137(6.1) | 66(2.9) | 123(5.5) | 80(3.5) | 231(10.3) | 541(24.1) | 753  (33.5) | 368  (16.4) | 497  (22.1) |
| Pitangueira et al, 2014 | Brazil | 502 | 213/289 | 7-14 | - | 64  (12.8) | - | CS | 14(2.8) | 50(10) | 35(7) | 29(5.8) | 134(26.7) | 265(52.8) | 300 (41.8) | 36(7.2) | 146 (29.1) |
| Mbowe et al, 2014 | Guatemala | 302 | 144/158 | 8-13 | - | 6(2) | - | CS | - | - | - | - | 37 (12.3) | 52 (17.2) | 131 (43.4) | 5 (1.7) | 6 (2.0) |
| Li et al, 2014 | China | 910 | 485/425 | 11-16 | 69(7.6) | - | - | CS | - | - | 53(5.8) | 16(1.8) | 205 (22.5) | 426 (46.8) | 88 (9.7) | 57(6.3) | 154 (16.9) |
| Gobato et al, 2014 | Brazil | 79 | 40/39 | 10-18 | 36(45.5) | - |  | CS | - | 36  (45.5) | 19 (24) | 17 (21.5) | - | - | - | - | - |
| Fadzlina1 et al, 2014 | Malaysia | 1014 | 387/627 | 13 | 26(2.6) | - | - | CS | - | 26(2.6) | 13(1.3) | 13(1.3) | 175(17.3) | 64(6.3) | 67(6.6) | 35(3.5) | 50(4.9) |
| Casavalle et al, 2014 | Argentina | 139 | 78/61 | 8-14 | - | 30 (21.6) | - | CS | - | 30 (21.6) | - | - | 77(55.4) | 41(29.5) | 44(31.7) | 2(1.5) | 35(25.2) |
| Yee et al, 2013 | Myanmar | 46 | 25/21 | 5-12 | 9(19.6) | - | - | CS | - | 9(19.6) | - | - | 25 (54.4) | 28 (60.9) | 6 (13.0) | 2 (4.3) | 4 (8.7) |
| Wang et al, 2013 | China | 2564 | 1279/1285 | 10-18 | 140(5.5) | 331  (12.9) | - | CS | - | 140(5.5) | 87(3.4) | 53(2.1) | 806(31.4) | 362(14.1) | 264  (10.3) | 323 (12.6) | 255(9.9) |
|  |  |  |  |  |  |  |  |  | 12(0.5) | 319(12.4) | 208(8.1) | 123(4.8) | 835(32.6) | 306(11.9) | 649(25.3) | 323(12.6 | 497(19.4) |
| Tandona et al, 2013 | India | 695 | 346/349 | 10–18 | 118(17) | 137  (19.7) | - | CS | 1(0.2) | 117  (16.8) | - | - | 273(39.3) | 190(27.3) | 258(37.) | 92  (13.2) | 97(14) |
| Sua´ rez-Ortego’n et al, 2013 | Colombia | 1461 | 718/743 | 10–16 | 18(1.2) | 37(2.5) | 124(8.5) | CS | 6(0.4) | 12(0.8) | 8(0.5) | 10(0.7) | 129(8.8) | 39(26.8) | 101(6.9) | 66(4.5) | 53(3.6) |
|  |  |  |  |  |  |  |  |  |  |  |  |  | 325(22.2) | 798(54.6) | 402(27.5) | 10(0.7) | 88(6) |
|  |  |  |  |  |  |  |  |  |  |  |  |  | 129(8.8) | 433(29.6) | 297(20.3) | 10(0.7) | 156(8.6) |
| Singh et al, 2013 | India | 1160 | 658/502 | 10‑18 | - | 31(2.67) | - | CS | 11(0.9) | 20(1.7) | 25(2.2) | 6(0.47) | 60 (5.66 | 124 (10.66) | 40 (3.44) | 70(6.3) | 32 (2.75) |
| Sewaybrickera et al, 2013 | Brazil | 65 | 32/33 | 10-18 | 18(27.7) | 19 (29.2) | - | CS | - | 18(27.6) | 7(10.7) | 11(11.9) | 17(27.7) | 18(27.7) | 18(27.7) | 17(27.7) | 18(27.7) |
|  |  |  |  |  |  |  |  | CS | - | 19 (29.2) | 8(10.7) | 11(11.9) | 18(27.7) | 19(29.2) | 19(29.2) | 18(27.7) | 19(29.2) |
| Sarrafzadegan et al, 2013 | Iran | 1992 | 1014/978 | - | 90(4.5) | - | 240(12.1) | CS | - | - | - | - | 179(9) | 496 (24.9) | 217(10.9) | 92(4.6) | 454(22.8) |
|  |  |  |  |  |  |  |  |  |  |  | 140(7) | 100(5.1) | 420(21) | 496(24.9) | 854(42.9 | 92(4.6) | 454(22.8 |
| Rizzo et l, 2013 | Brazil | 321 | 147/174 | 10-16 | 59 (18.3) | - | - | CS | - | 59 (18.3) | 27(8.3) | 32(10) | 177(55) | 114(35.5) | 6(18.5) | 7(2) | 68(21) |
| Qorbani et al,2013 | Iran | 3565 | 1793/1772 | 10-18 | 91(2.6) | - | - | CS | - | 40(1.2) | 51(1.4) | - | - | - | - | - | - |
| Khashayar et al, 2013 | Iran | 5738 | 2863/ 2875 | 10-18 | 144(2.5) | - | - | CS | 67(1.1) | 77(1.4) | - | - | 935(16.3) | 1431(24.9) | 372(6.5) | 697  (12.1) | 307(5.4) |
| Andrabi et al, 2013 | India | 758 | 385/ 373 | 8-18 | - | 29(3.8) | - | CS | 3(0.4) | 26(3.4) | 15(2) | 14(1.8) | 34(4.5) | 33 (4.4) | 29 (3.8) | 10(1.3) | - |
| Xu et al, 2012 | China | 8764 | 4495/ 4269 | 7-11 | 52(0.6) | - | - | CS | 3(0.05) | 49(0.55) | 30(0.35) | 22(0.25) | 1195(13.6) | 460(5.2) | 346(3.9) | 188 (2.1) | 161(1.8) |
| Saffari et al, 2012 | Iran | 100 | 42/58 | 6-16 | - | 63(63) | - | CS | - | 63(63) | 24(24) | 39(39) | 81 | 70 | 74 | 12 | 36 |
|  |  |  |  |  |  |  |  |  | - |  | 19 | 20 | 81 | 30 | 65 | 12 | 34 |
| Nasreddine e al, 2012 | Lebanon | 263 | 112/115 | - | 24(9.1) | 26(9.9) | - | CS | 1(0.4) | 23(8.7) | - | - | 133(50.6) | 101(38.4) | 28(10.6) | 13(4.9) | 32(12.2) |
| Mehrkash et al, 2012 | Iran | 450 | 225/225 | 15-18 | - | 15(3.3) | - | CS | 4(0.9) | 11(2.4) | 11(2.4) | 4(0.9) | 19(4.2) | 52(11.6) | 180(33.3) | 56(12.4) | 22(4.9) |
| Jamoussi t al, 2012 | Tunisia | 186 | 49/137 | 6-18 | 64(34.4) | - | - | CS | - | 64(34.4) | 44(23.7) | 20(10.7) | 186(100) | 50(27) | 28(15) | 95(51) | 52(28) |
| Cua et al, 2012 | Philippines | 350 | 206/144 | 10-18 | 67(19) | - | - | CS | - | 67(19) | 41(11.7) | 26(7.3) | 343(98) | 60(17) | 84(24) | 42(12) | 88(25) |
| Costa et al, 2012 | Brazil | 121 | 62/59 | 10-14 | 48(39.7) | 62(51.2 | (74.4) | CS | - | 48(39.7) | - | - | 81 | 54.5 | 16.5 | 7.4 | 54.5 |
|  |  |  |  |  |  |  |  |  | - | 62(51.2 | - | - | 81 | 54.5 | 34.7 | 1.7 | 76 |
|  |  |  |  |  |  |  |  |  | - | 90(74.4) | - | - | 96.7 | 92.6 | 40.5 | 1.7 | 76 |
| Chen et al, 2012 | China | 3814 | - | 10-18 | 372(9.8) | - | - | CS | 4(0.2) | 368(9.6) | - | - | - | - | 1724(45) | 490(13) | - |
| Hassan et al, 2011 | Egypt | 462 | 144/288 | 7-18 | - | - | 184(39.7) | CS | - | 184(39.7) | - | - | 396(85.7) | 148(32) | 198(42.9) | 64(13.9) | 140(30.3) |
| Panamonta et al, 2010 | Thailand | 186 |  | 10-15 | 6 (3.2) | - | - | CS | - | 6 (3.2) | - | - | - | 19 (10.2) | 52 (28.0) | 2 (1.1) | 16 (8.6) |
| Liu e al, 2010 | China | 1844 | 938/906 | 7-14 | - | 121(6.6) | - | CS | 35(1.9) | 86(4.7) | 53(2.9) | 68(3.7) | 432(23.4) | 292( 15.8) | 296(16.1) | 3(0.2) | 434(23.5) |
| Khader et al, 2010 | Jordan | 512 | 235/277 | 10–18 | 11(2.1) | - | - | CS | - | - | - | - | 30(5.8) | 134(26.1) | 88(17.2) | 37(7.2) | 32(6.2) |
| Juárez-López etal, 2010 | Mexico | 466 | 272/194 | 11-13 | 93(20) | - | - | CS | - | 93(20) | 57(12.2) | 36(7.8) | 228(49) | 322(69) | 135(29) | 19(4) | 61(13) |
| Hirschler et al, 2010 | Argentina | 1009 | 508/501 | 6-14 | - | 57(5.8) | - | CS | 3(0.4) | 54(5.4) | 27(2.8 | 30(3) | 279(27.6) | 199(19.7) | 130(12.9) | 8(0.8) | 86(8.5) |
| Ella et al, 2010 | Egypt | 4250 | 1806/2444 | 10-18 | - | 308(7.2) | - | CS | - | - | 134(3.1) | 174(4.1) | 855(20) | 520(24) | 482(22) | 179(4) | 1082(25.5) |
| Afkhami-Ardekani et al, 2010 | Iran | 932 | 402/530 | 10–19 | 75(8) | 63(6.7) | - | CS | - | - | - | - | - | - | - | - | - |
| Seki1 et al, 2009 | Brazil | 2170 | 1103/1067 | 6-16 | - | 78(3.6) | - | CS | 6(0.3) | 72(3.3) | 46(2.1) | 32(1.5) | 243(11.2 ) | 938(43.2) | 139(6.4) | 0.6(13) | 9.8(213) |
| Salem et al, 2009 | Iran | 1221 | 0/1221 | 11-18 | - | 48(3.9) | - | CS | - | - | - | 48(3.9) | 15(1.2) | 54(44.7) | 193(15.8 | 97(7.9) | 18(1.5) |
| Mirhosseini et al, 2009 | Iran | 622 | 0/622 | 15-17 | - | 40(6.5) | - | CS | 30(4.8) | 10(1.7) | - | 40(6.5) | 23(3.7) | 355(57) | 152(24.5) | 104(16.7) | 38(6.1) |
| Matsha et al, 2009 | S.Africa | 1272 | 496/776 | 10-16 | 24(1.9) | 83(6.5) | - | CS | 28(2.2) | 55(4.3) | 40(3.1) | 43(3.4) | 126(9.9) | 614(48.3) | 118(9.3) | 53(4.2) | 119(9.3) |
|  |  |  |  |  |  |  |  | CS | 12(0.95) | 12(0.95) | 17(1.3) | 7(0.6) | 138(10.8 | 614(48.3) | 52(4.1) | 53(4.2) | 86(6.8) |
| Li et al, 2008 | China | 2761 | 1478/1283 | 15-19 | - | - | 102(3·7) | CS | 61(2.2) | 41(1.5) | 50(1.8) | 52(1.9) | 105(3·8) | 1485(53·8) | 541(19·6) | 22(0·8) | 503(18·2) |
| Caceres et al, 2008 | Bolivia | 61 | 30/31 | 5-18 | - | 22(36) | - | CS | - | 22(36) | 12(19.7) | 10(16.3) | 61(100) | 34(55.7) | 26(42.6) | 5(8.2) | 15(24.5) |
| Singh et al, 2007 | India | 1083 | 571/512 | 12-17 | - | 46(4.2) | - | CS | 19(1.7) | 27(2.5) | 18(1.6) | 28(2.6) | 43(4) | 279(25.8) | 221(20.4) | 54(5) | 85(7.8) |
| Kelishadi et al, 2006 | Iran | 4811 | 2248/2563 | 6-18 | - | 678(14) | - | CS | - | - | - | - | 1107(23) | 3464(72) | 1828(38) | 193(4) | 337(7) |
| Esmaillzadeh et al, 2006 | Iran | 3036 | 1413/1623 | 10-19 | - | 307(10.1) | - | CS | 119(3.9) | 188(6.2) | 146(4.8) | 161(5.3) | 304(10) | 1299(42.8) | 1139(37.5) | 18(0.6) | 723(23.8) |
| Rodríguez-Morán et al, 2004 | Mexico | 965 | 499/466 | 10-18 | - | 63(6.5) | - | CS | - | - | 23(2.4) | 40(4.1) | 267(27.7) | 201(20.8) | 92(9.5) | 74(7.7) | 69(7.1) |
| Damak et al, 2015 | Tunisia | 51 | 28/23 | 15-18 | 11(21.6) | - | - | CS | - | 11(21.6) | 6(11.8) | 5(9.8) | 6(11.8) | 58.8 | 9.8 | - | 27.4 |
| Ramı´rez-Ve´ lez et al, 2016 | Colombia | 1922 | 877/1045 | 9-17 | 6(0.3) | 119(6.2) | 211(11) | CS | 3(0.15) | 3(0.15) | 1(0.04) | 5(0.26) | - | - | - | - | - |
|  |  |  |  |  |  |  |  | CS | 78(4) | 41(2.2) | 49(2.5) | 71(3.7) | - | - | - | - | - |
|  |  |  |  |  |  |  |  | CS | 133(7) | 78(4) | 86(4.5) | 125(6.5) | - | - | - | - | - |
| Bortoloti et al, 2015 | Brazil | 683 | 301/382 | 11-17 | - | 37(5.4) | - | CS | - | - | - | - | 24(3.5) | 305(44.7) | 127(18.6) | 4(0.6) | 48(7) |
