# Supplementary material for: Metabolic syndrome among children and adolescents in low and middle income countries: a systematic review and meta-analysis
Source: Diabetol Metab Syndr. 2020 Oct 27;12:93. doi: 10.1186/s13098-020-00601-8 (PMC7590497; doi:10.1186/s13098-020-00601-8)
Supplement: Supplementary file 2 — Additional file 2. Critical appraisal of the included studies. [file 13098_2020_601_MOESM2_ESM.docx]

**Critical appraisal of cross sectional studies**

| Author, year | Q1 | | | | Q2 | | | | Q3 | | | | | Q4 | | | | | Q5 | | | | Q6 | | | | Q7 | | | | Q8 | | | | Overall quality result |
| --- | --- | --- | --- | --- | --- | --- | --- | --- | --- | --- | --- | --- | --- | --- | --- | --- | --- | --- | --- | --- | --- | --- | --- | --- | --- | --- | --- | --- | --- | --- | --- | --- | --- | --- | --- |
|  | Y | N | U | NA | Y | N | U | NA | | Y | N | U | NA | | Y | N | U | NA | Y | N | U | NA | Y | N | U | NA | Y | N | U | NA | Y | N | U | NA |  |
| Zhu et al, 2020 | √ |  |  |  | √ |  |  |  | | √ |  |  |  | | √ |  |  |  |  |  |  | √ |  |  |  | √ | √ |  |  |  | √ |  |  |  | 6/8(75%) |
| Mahajan et al, 2020 | √ |  |  |  | √ |  |  |  | | √ |  |  |  | | √ |  |  |  | √ |  |  |  | √ |  |  |  | √ |  |  |  | √ |  |  |  | 8/8(100%) |
| Dejavitte et al,2020 | √ |  |  |  | √ |  |  |  | | √ |  |  |  | | √ |  |  |  | √ |  |  |  | √ |  |  |  | √ |  |  |  | √ |  |  |  | 8/8(100%) |
| Bekele et al,2020 | √ |  |  |  | √ |  |  |  | | √ |  |  |  | | √ |  |  |  |  |  |  | √ |  |  |  | √ | √ |  |  |  | √ |  |  |  | 6/8(75%) |
| Ahmadi et al,2020 | √ |  |  |  | √ |  |  |  | | √ |  |  |  | | √ |  |  |  | √ |  |  |  | √ |  |  |  | √ |  |  |  | √ |  |  |  | 8/8(100%) |
| Zhao et al, 2019 | √ |  |  |  | √ |  |  |  | | √ |  |  |  | | √ |  |  |  | √ |  |  |  | √ |  |  |  | √ |  |  |  | √ |  |  |  | 8/8(100%) |
| Zhang et al,2019 | √ |  |  |  | √ |  |  |  | | √ |  |  |  | | √ |  |  |  |  |  |  | √ |  |  |  | √ | √ |  |  |  | √ |  |  |  | 6/8(75%) |
| Wang et al,2019 | √ |  |  |  | √ |  |  |  | | √ |  |  |  | | √ |  |  |  | √ |  |  |  | √ |  |  |  | √ |  |  |  | √ |  |  |  | 8/8(100%) |
| Oliveira et al,2019 | √ |  |  |  | √ |  |  |  | | √ |  |  |  | | √ |  |  |  |  |  |  | √ |  |  |  | √ | √ |  |  |  | √ |  |  |  | 6/8(75%) |
| Suebsamran et al,2018 | √ |  |  |  | √ |  |  |  | | √ |  |  |  | | √ |  |  |  |  |  |  | √ |  |  |  | √ | √ |  |  |  | √ |  |  |  | 6/8(75%) |
| Gupta et al, 2018 | √ |  |  |  | √ |  |  |  | | √ |  |  |  | | √ |  |  |  |  |  |  | √ |  |  |  | √ | √ |  |  |  | √ |  |  |  | 6/8(75%) |
| Dos Santos et al, 2018 | √ |  |  |  | √ |  |  |  | | √ |  |  |  | | √ |  |  |  |  |  |  | √ |  |  |  | √ | √ |  |  |  | √ |  |  |  | 6/8(75%) |
| Andaki et al,2018 | √ |  |  |  | √ |  |  |  | | √ |  |  |  | | √ |  |  |  |  |  |  | √ |  |  |  | √ | √ |  |  |  | √ |  |  |  | 6/8(75%) |
| Sekokotla et al, 2017 | √ |  |  |  | √ |  |  |  | | √ |  |  |  | | √ |  |  |  |  |  |  | √ |  |  |  | √ | √ |  |  |  | √ |  |  |  | 6/8(75%) |
| Cornejo-Monthedoro et al,2017 | √ |  |  |  | √ |  |  |  | | √ |  |  |  | | √ |  |  |  | √ |  |  |  | √ |  |  |  | √ |  |  |  | √ |  |  |  | 8/8(100%) |
| Wang et al, 2016 | √ |  |  |  | √ |  |  |  | | √ |  |  |  | | √ |  |  |  |  |  |  | √ |  |  |  | √ | √ |  |  |  | √ |  |  |  | 6/8(75%) |
| Suarez-Ortegón et al, 2016 | √ |  |  |  | √ |  |  |  | | √ |  |  |  | | √ |  |  |  |  |  |  | √ |  |  |  | √ | √ |  |  |  | √ |  |  |  | 6/8(75%) |
| Rinaldi et al, 2016 | √ |  |  |  | √ |  |  |  | | √ |  |  |  | | √ |  |  |  | √ |  |  |  | √ |  |  |  | √ |  |  |  | √ |  |  |  | 8/8(100%) |
| Kuschnir et al, 2016 | √ |  |  |  | √ |  |  |  | | √ |  |  |  | | √ |  |  |  |  |  |  | √ |  |  |  | √ | √ |  |  |  | √ |  |  |  | 6/8(75%) |
| Karandish et al, 2016 | √ |  |  |  | √ |  |  |  | | √ |  |  |  | | √ |  |  |  | √ |  |  |  | √ |  |  |  | √ |  |  |  | √ |  |  |  | 8/8(100%) |
| de Carvalho et al, 2016 | √ |  |  |  | √ |  |  |  | | √ |  |  |  | | √ |  |  |  |  |  |  | √ |  |  |  | √ | √ |  |  |  | √ |  |  |  | 6/8(75%) |
| Vukovic et al, 2015 | √ |  |  |  | √ |  |  |  | | √ |  |  |  | | √ |  |  |  |  |  |  | √ |  |  |  | √ | √ |  |  |  | √ |  |  |  | 6/8(75%) |
| Rosini et al, 2015 | √ |  |  |  | √ |  |  |  | | √ |  |  |  | | √ |  |  |  | √ |  |  |  | √ |  |  |  | √ |  |  |  | √ |  |  |  | 8/8(100%) |
| Medina et al, 2015 | √ |  |  |  | √ |  |  |  | | √ |  |  |  | | √ |  |  |  |  |  |  | √ |  |  |  | √ | √ |  |  |  | √ |  |  |  | 6/8(75%) |
| Bhat et al, 2015 | √ |  |  |  | √ |  |  |  | | √ |  |  |  | | √ |  |  |  |  |  |  | √ |  |  |  | √ | √ |  |  |  | √ |  |  |  | 6/8(75%) |
| Bhalavi et al, 2015 | √ |  |  |  | √ |  |  |  | | √ |  |  |  | | √ |  |  |  |  |  |  | √ |  |  |  | √ | √ |  |  |  | √ |  |  |  | 6/8(75%) |
| Reyes, et al, 2014 | √ |  |  |  | √ |  |  |  | | √ |  |  |  | | √ |  |  |  |  |  |  | √ |  |  |  | √ | √ |  |  |  | √ |  |  |  | 6/8(75%) |
| Tavares Giannini et a, 2014 | √ |  |  |  | √ |  |  |  | | √ |  |  |  | | √ |  |  |  | √ |  |  |  | √ |  |  |  | √ |  |  |  | √ |  |  |  | 8/8(100%) |
| Rerksuppaphol et al, 2014 | √ |  |  |  | √ |  |  |  | | √ |  |  |  | | √ |  |  |  |  |  |  | √ |  |  |  | √ | √ |  |  |  | √ |  |  |  | 6/8(75%) |
| Rashidi et al, 2014 | √ |  |  |  | √ |  |  |  | | √ |  |  |  | | √ |  |  |  |  |  |  | √ |  |  |  | √ | √ |  |  |  | √ |  |  |  | 6/8(75%) |
| Pitangueira et al, 2014 | √ |  |  |  | √ |  |  |  | | √ |  |  |  | | √ |  |  |  |  |  |  | √ |  |  |  | √ | √ |  |  |  | √ |  |  |  | 6/8(75%) |
| Mbowe et al, 2014 | √ |  |  |  | √ |  |  |  | | √ |  |  |  | | √ |  |  |  | √ |  |  |  | √ |  |  |  | √ |  |  |  | √ |  |  |  | 8/8(100%) |
| Li et al, 2014 | √ |  |  |  | √ |  |  |  | | √ |  |  |  | | √ |  |  |  | √ |  |  |  | √ |  |  |  | √ |  |  |  | √ |  |  |  | 8/8(100%) |
| Gobato et al, 2014 | √ |  |  |  | √ |  |  |  | | √ |  |  |  | | √ |  |  |  |  |  |  | √ |  |  |  | √ | √ |  |  |  | √ |  |  |  | 6/8(75%) |
| Fadzlina1 et al, 2014 | √ |  |  |  | √ |  |  |  | | √ |  |  |  | | √ |  |  |  | √ |  |  |  | √ |  |  |  | √ |  |  |  | √ |  |  |  | 8/8(100%) |
| Casavalle et al, 2014 | √ |  |  |  | √ |  |  |  | | √ |  |  |  | | √ |  |  |  |  |  |  | √ |  |  |  | √ | √ |  |  |  | √ |  |  |  | 6/8(75%) |
| Yee et al, 2013 | √ |  |  |  | √ |  |  |  | | √ |  |  |  | | √ |  |  |  |  |  |  | √ |  |  |  | √ | √ |  |  |  | √ |  |  |  | 6/8(75%) |
| Wang et al, 2013 | √ |  |  |  | √ |  |  |  | | √ |  |  |  | | √ |  |  |  |  |  |  | √ |  |  |  | √ | √ |  |  |  | √ |  |  |  | 6/8(75%) |
| Tandona et al, 2013 | √ |  |  |  | √ |  |  |  | | √ |  |  |  | | √ |  |  |  |  |  |  | √ |  |  |  | √ | √ |  |  |  | √ |  |  |  | 6/8(75%) |
| Sua´ rez-Ortego’n et al, 2013 | √ |  |  |  | √ |  |  |  | | √ |  |  |  | | √ |  |  |  |  |  |  | √ |  |  |  | √ | √ |  |  |  | √ |  |  |  | 6/8(75%) |
| Singh et al, 2013 | √ |  |  |  | √ |  |  |  | | √ |  |  |  | | √ |  |  |  | √ |  |  |  | √ |  |  |  | √ |  |  |  | √ |  |  |  | 8/8(100%) |
| Sewaybrickera et al, 2013 | √ |  |  |  | √ |  |  |  | | √ |  |  |  | |  |  | √ |  |  |  |  | √ |  |  |  | √ | √ |  |  |  | √ |  |  |  | 5/8(62.5%) |
| Sarrafzadegan et al, 2013 | √ |  |  |  | √ |  |  |  | | √ |  |  |  | | √ |  |  |  |  |  |  | √ |  |  |  | √ | √ |  |  |  | √ |  |  |  | 6/8(75%) |
| Rizzo et l, 2013 | √ |  |  |  | √ |  |  |  | | √ |  |  |  | | √ |  |  |  |  |  |  | √ |  |  |  | √ | √ |  |  |  | √ |  |  |  | 6/8(75%) |
| Qorbani et al,2013 | √ |  |  |  | √ |  |  |  | | √ |  |  |  | | √ |  |  |  |  |  |  | √ |  |  |  | √ | √ |  |  |  | √ |  |  |  | 6/8(75%) |
| Khashayar et al, 2013 | √ |  |  |  | √ |  |  |  | | √ |  |  |  | | √ |  |  |  | √ |  |  |  | √ |  |  |  | √ |  |  |  | √ |  |  |  | 8/8(100%) |
| Andrabi et al, 2013 | √ |  |  |  | √ |  |  |  | | √ |  |  |  | | √ |  |  |  | √ |  |  |  | √ |  |  |  | √ |  |  |  | √ |  |  |  | 8/8(100%) |
| Xu et al, 2012 | √ |  |  |  | √ |  |  |  | | √ |  |  |  | | √ |  |  |  | √ |  |  |  | √ |  |  |  | √ |  |  |  | √ |  |  |  | 8/8(100%) |
| Saffari et al, 2012 | √ |  |  |  | √ |  |  |  | | √ |  |  |  | |  |  | √ |  |  |  |  | √ |  |  |  | √ | √ |  |  |  | √ |  |  |  | 5/8(62.5%) |
| Nasreddine e al, 2012 | √ |  |  |  | √ |  |  |  | | √ |  |  |  | | √ |  |  |  |  |  |  | √ |  |  |  | √ | √ |  |  |  | √ |  |  |  | 6/8(75%) |
| Mehrkash et al, 2012 | √ |  |  |  | √ |  |  |  | | √ |  |  |  | | √ |  |  |  |  |  |  | √ |  |  |  | √ | √ |  |  |  | √ |  |  |  | 6/8(75%) |
| Jamoussi t al, 2012 | √ |  |  |  | √ |  |  |  | | √ |  |  |  | |  |  | √ |  |  |  |  | √ |  |  |  | √ | √ |  |  |  | √ |  |  |  | 5/8(62.5%) |
| Cua et al, 2012 | √ |  |  |  | √ |  |  |  | | √ |  |  |  | | √ |  |  |  |  |  |  | √ |  |  |  | √ | √ |  |  |  | √ |  |  |  | 6/8(75%) |
| Costa et al, 2012 | √ |  |  |  | √ |  |  |  | | √ |  |  |  | | √ |  |  |  |  |  |  | √ |  |  |  | √ | √ |  |  |  | √ |  |  |  | 6/8(75%) |
| Chen et al, 2012 | √ |  |  |  | √ |  |  |  | | √ |  |  |  | | √ |  |  |  |  |  |  | √ |  |  |  | √ | √ |  |  |  | √ |  |  |  | 6/8(75%) |
| Hassan et al, 2011 | √ |  |  |  | √ |  |  |  | | √ |  |  |  | | √ |  |  |  |  |  |  | √ |  |  |  | √ | √ |  |  |  | √ |  |  |  | 6/8(75%) |
| Panamonta et al, 2010 | √ |  |  |  | √ |  |  |  | | √ |  |  |  | | √ |  |  |  |  |  |  | √ |  |  |  | √ | √ |  |  |  | √ |  |  |  | 6/8(75%) |
| Liu e al, 2010 | √ |  |  |  | √ |  |  |  | | √ |  |  |  | | √ |  |  |  |  |  |  | √ |  |  |  | √ | √ |  |  |  | √ |  |  |  | 6/8(75%) |
| Khader et al, 2010 | √ |  |  |  | √ |  |  |  | | √ |  |  |  | | √ |  |  |  |  |  |  | √ |  |  |  | √ | √ |  |  |  | √ |  |  |  | 6/8(75%) |
| Juárez-López etal, 2010 | √ |  |  |  | √ |  |  |  | | √ |  |  |  | | √ |  |  |  |  |  |  | √ |  |  |  | √ | √ |  |  |  | √ |  |  |  | 6/8(75%) |
| Hirschler et al, 2010 | √ |  |  |  | √ |  |  |  | | √ |  |  |  | | √ |  |  |  |  |  |  | √ |  |  |  | √ | √ |  |  |  | √ |  |  |  | 6/8(75%) |
| Ella et al, 2010 | √ |  |  |  | √ |  |  |  | | √ |  |  |  | | √ |  |  |  | √ |  |  |  | √ |  |  |  | √ |  |  |  | √ |  |  |  | 8/8(100%) |
| Afkhami-Ardekani et al, 2010 | √ |  |  |  | √ |  |  |  | | √ |  |  |  | |  |  | √ |  |  |  |  | √ |  |  |  | √ | √ |  |  |  | √ |  |  |  | 5/8(62.5%) |
| Seki et al, 2009 | √ |  |  |  | √ |  |  |  | | √ |  |  |  | | √ |  |  |  | √ |  |  |  | √ |  |  |  | √ |  |  |  | √ |  |  |  | 8/8(100%) |
| Salem et al, 2009 | √ |  |  |  | √ |  |  |  | | √ |  |  |  | | √ |  |  |  | √ |  |  |  | √ |  |  |  | √ |  |  |  | √ |  |  |  | 8/8(100%) |
| Mirhosseini et al, 2009 | √ |  |  |  | √ |  |  |  | | √ |  |  |  | |  |  | √ |  |  |  |  | √ |  |  |  | √ | √ |  |  |  | √ |  |  |  | 5/8(62.5%) |
| Matsha et al, 2009 | √ |  |  |  | √ |  |  |  | | √ |  |  |  | |  |  | √ |  |  |  |  | √ |  |  |  | √ | √ |  |  |  | √ |  |  |  | 5/8(62.5%) |
| Li et al, 2008 | √ |  |  |  | √ |  |  |  | | √ |  |  |  | | √ |  |  |  |  |  |  | √ |  |  |  | √ | √ |  |  |  | √ |  |  |  | 6/8(75%) |
| Caceres et al, 2008 | √ |  |  |  | √ |  |  |  | | √ |  |  |  | | √ |  |  |  |  |  |  | √ |  |  |  | √ | √ |  |  |  | √ |  |  |  | 6/8(75%) |
| Singh et al, 2007 | √ |  |  |  | √ |  |  |  | | √ |  |  |  | |  |  | √ |  |  |  |  | √ |  |  |  | √ | √ |  |  |  | √ |  |  |  | 5/8(62.5%) |
| Kelishadi et al, 2006 | √ |  |  |  | √ |  |  |  | | √ |  |  |  | | √ |  |  |  |  |  |  | √ |  |  |  | √ | √ |  |  |  | √ |  |  |  | 6/8(75%) |
| Esmaillzadeh et al, 2006 | √ |  |  |  | √ |  |  |  | | √ |  |  |  | | √ |  |  |  |  |  |  | √ |  |  |  | √ | √ |  |  |  | √ |  |  |  | 6/8(75%) |
| Rodríguez-Morán et al, 2004 | √ |  |  |  | √ |  |  |  | | √ |  |  |  | | √ |  |  |  |  |  |  | √ |  |  |  | √ | √ |  |  |  | √ |  |  |  | 6/8(75%) |
| Damak et al, 2015 | √ |  |  |  | √ |  |  |  | | √ |  |  |  | | √ |  |  |  |  |  |  | √ |  |  |  | √ | √ |  |  |  | √ |  |  |  | 6/8(75%) |
| Ramı´rez-Ve´ lez et al, 2016 | √ |  |  |  | √ |  |  |  | | √ |  |  |  | | √ |  |  |  |  |  |  | √ |  |  |  | √ | √ |  |  |  | √ |  |  |  | 6/8(75%) |
| Bortoloti et al, 2015 | √ |  |  |  | √ |  |  |  | | √ |  |  |  | | √ |  |  |  |  |  |  | √ |  |  |  | √ | √ |  |  |  | √ |  |  |  | 6/8(75%) |

****Y=yes, N=no, U=unclear, NA=not applicable, <60%=low, 60-80%=medium, >80%=high quality***
