# Supplementary material for: Metabolic syndrome among children and adolescents in low and middle income countries: a systematic review and meta-analysis
Source: Diabetol Metab Syndr. 2020 Oct 27;12:93. doi: 10.1186/s13098-020-00601-8 (PMC7590497; doi:10.1186/s13098-020-00601-8)
Supplement: Supplementary file 3 — Additional file 3. List of excluded studies. [file 13098_2020_601_MOESM3_ESM.docx]

List of excluded studies

Twenty five studies were excluded due to the following exclusion criteria: different study population [1-5], no full test [6-9], unclear diagnostic criteria [10-17], letter to editor [18], written in non-English language [19, 20], and different study design [21-25]

1. Castillo EH, Borges G, Talavera JO, Orozco R, Vargas-Alemán C, Huitrón-Bravo G, Diaz-Montiel JC, Castañón S, Salmerón J: **Body mass index and the prevalence of metabolic syndrome among children and adolescents in two Mexican populations**. *Journal of Adolescent Health* 2007, **40**(6):521-526.

2. Ahmadi A, Gharipour M, Nouri F, Sarrafzadegan N: **Metabolic syndrome in Iranian youths: a population-based study on junior and high schools students in rural and urban areas**. *Journal of diabetes research* 2013, **2013**:738485.

3. Kuschnir FC, Felix MMR, Caetano Kuschnir MC, Bloch KV, Azevedo de Oliveira Costa Jordão E, Solé D, Ledo Alves da Cunha AJ, Szklo M: **Severe asthma is associated with metabolic syndrome in Brazilian adolescents**. *The Journal of allergy and clinical immunology* 2018, **141**(5):1947-1949.e1944.

4. Dabbaghmanesh MH, Naderi T, Akbarzadeh M, Tabatabaee H: **Metabolic syndrome in Iranian adolescents with polycystic ovary syndrome**. *International journal of adolescent medicine and health* 2017, **31**(4).

5. Bjerregaard-Andersen M, Hansen L, da Silva LI, Joaquím LC, Hennild DE, Christiansen L, Aaby P, Benn CS, Christensen K, Sodemann M *et al*: **Risk of metabolic syndrome and diabetes among young twins and singletons in Guinea-Bissau**. *Diabetes care* 2013, **36**(11):3549-3556.

6. Cheng X, Wang H, Yuan B, Guan P, Wang L: **Prevalence of metabolic syndrome and its family factors for children and adolescents in Chongqing City in 2014**. *Wei sheng yan jiu= Journal of hygiene research* 2017, **46**(4):557-562.

7. Aghbar A, Tayem M, Nana A, Qamhia D, Musmar H, Yahia D: **Prevalence of metabolic syndrome among school children aged 6–18 years in Ein Al-Helwa Palestinian Refugee Camp, Lebanon: a cross-sectional study**. *The Lancet* 2019, **393**:S2.

8. Iamopas O, Chongviriyaphan N, Suthutvoravut U: **Metabolic syndrome in obese Thai children and adolescents**. *Journal of the Medical Association of Thailand= Chotmaihet thangphaet* 2011, **94**:S126-132.

9. Rerksuppaphol S, Rerksuppaphol L: **Metabolic Syndrome in Obese Thai Children: Defined Using Modified 'The National Cholesterol Education Program/Adult Treatment Panel III' Criteria**. *Journal of the Medical Association of Thailand = Chotmaihet thangphaet* 2015, **98 Suppl 10**:S88-95.

10. Kimani-Murage EW, Kahn K, Pettifor JM, Tollman SM, Dunger DB, Gómez-Olivé XF, Norris SA: **The prevalence of stunting, overweight and obesity, and metabolic disease risk in rural South African children**. *BMC public health* 2010, **10**(1):158.

11. Barzin M, Hosseinpanah F, Saber H, Sarbakhsh P, Nakhoda K, Azizi F: **Gender Differences Time Trends for Metabolic Syndrome and Its Components among Tehranian Children and Adolescents**. *Cholesterol* 2012, **2012**:804643.

12. Barbalho SM, Oshiiwa M, Fontana LCS, Finalli EFR, Paiva Filho ME, Spada APM: **Metabolic syndrome and atherogenic indices in school children: A worrying panorama in Brazil**. *Diabetes & Metabolic Syndrome: Clinical Research & Reviews* 2017, **11**:S397-S401.

13. Mirmiran P, Sherafat-Kazemzadeh R, Farahani SJ, Asghari G, Niroomand M, Momenan A, Azizi F: **Performance of different definitions of metabolic syndrome for children and adolescents in a 6-year follow-up: Tehran Lipid and Glucose Study (TLGS)**. *Diabetes research and clinical practice* 2010, **89**(3):327-333.

14. Banaś I, Lewek P, Kardas P: **In which group of children and adolescents should a family doctor look for metabolic syndrome?** *Family Medicine & Primary Care Review* 2016(3):217-220.

15. Kelishadi R, Razaghi EM, Gouya MM, Ardalan G, Gheiratmand R, Delavari A, Motaghian M, Ziaee V, Siadat ZD, Majdzadeh R: **Association of physical activity and the metabolic syndrome in children and adolescents: CASPIAN Study**. *Hormone research in paediatrics* 2007, **67**(1):46-52.

16. Heshmat R, Hemati Z, Payab M, Hamzeh SS, Motlagh ME, Shafiee G, Taheri M, Ziaodini H, Qorbani M, Kelishadi R: **Prevalence of different metabolic phenotypes of obesity in Iranian children and adolescents: the CASPIAN V study**. *Journal of Diabetes & Metabolic Disorders* 2018, **17**(2):211-221.

17. Ghosh A: **Factor analysis of risk variables associated with metabolic syndrome in Asian Indian adolescents**. *American Journal of Human Biology* 2007, **19**(1):34-40.

18. Kapil U, Kaur S: **Prevalence of pediatrics metabolic syndrome (PMS) amongst children in the age group of 6-18 years belonging to high income group residing in national capital territory (NCT) of Delhi**. *Indian journal of pediatrics* 2010, **77**(9):1041.

19. Romero-Velarde E, Aguirre-Salas LM, Álvarez-Román YA, Vásquez-Garibay EM, Casillas-Toral E, Fonseca-Reyes S: **Prevalence of metabolic syndrome and associated factors in children and adolescents with obesity**. *Revista Médica del Instituto Mexicano del Seguro Social* 2016, **54**(5):568-575.

20. Peña-Espinoza BI, Granados-Silvestre M, Sánchez-Pozos K, Ortiz-López MG, Menjivar M: **Metabolic syndrome in Mexican children: Low effectiveness of diagnostic definitions**. *Endocrinologia, diabetes y nutricion* 2017, **64**(7):369-376.

21. Zaki ME, Mohamed SK, Bahgat KA, Kholoussi SM: **Metabolic syndrome components in obese Egyptian children**. *Annals of Saudi medicine* 2012, **32**(6):603-610.

22. Asghari G, Yuzbashian E, Mirmiran P, Mahmoodi B, Azizi F: **Fast food intake increases the incidence of metabolic syndrome in children and adolescents: Tehran lipid and glucose study**. *PloS one* 2015, **10**(10):e0139641.

23. Mansour M, Nassef YE, Shady MA, Aziz AA, El Malt HA: **Metabolic syndrome and cardiovascular risk factors in obese adolescent**. *Open Access Macedonian Journal of Medical Sciences* 2016, **4**(1):118.

24. Mirmiran P, Yuzbashian E, Asghari G, Hosseinpour-Niazi S, Azizi F: **Consumption of sugar sweetened beverage is associated with incidence of metabolic syndrome in Tehranian children and adolescents**. *Nutrition & metabolism* 2015, **12**(1):25.

25. Hooshmand F, Asghari G, Yuzbashian E, Mahdavi M, Mirmiran P, Azizi F: **Modified Healthy Eating Index and Incidence of Metabolic Syndrome in Children and Adolescents: Tehran Lipid and Glucose Study**. *The Journal of pediatrics* 2018, **197**:134-139.e132.
